# Differences of patient empowerment between elective and contracted physicians in internal medicine in Austria: a quantitative content analysis

**DOI:** 10.1186/s12913-025-12635-7

**Published:** 2025-03-31

**Authors:** Bettina Simetsberger, Manfred Pferzinger, Martin K. J. Waiguny

**Affiliations:** 1 IMC University of Applied Sciences Krems, Krems, Austria; 2 Medizinische Informatik und Technik, UMIT - Private Universität für Gesundheitswissenschaften, Hall in Tyrol, Austria; 3 Oberösterreichische Gesundheitsholding GmbH, Linz, Austria

**Keywords:** Patient empowerment, Elective and contracted physicians, Physician-patient relationship, Time, Physician rating platform

## Abstract

**Background:**

Physicians impact and promote patient empowerment at various levels. Elective or contracted physicians uniquely provide specialised ambulatory care in Austria. The difference between the two groups is due to the contractual relationship with the social health insurance providers. This paper researches whether there are differences in the promotion of patient empowerment between elective and contracted physicians in internal medicine in Vienna based on four dimensions of patient empowerment.

**Methods:**

A quantitative content analysis analyses the comments on DocFinder.at to identify the differences between the two groups. A deductive-inductive approach based on literature and comments from DocFinder.at led to a codebook with seven categories and forty-eight variables. We collected a total of 1,153 comments, and 912 of them are relevant for further analysis. Differences between elective and contracted physicians became evident through defined dimensions of patient empowerment.

**Results:**

In the health literacy dimension, elective physicians apply relationship-relevant factors more effectively. They are also more successful in ensuring that patients feel adequately informed. No significant differences were found in the dimension of shared decision-making, but the discussion of treatment options correlated strongly with the amount of time spent by a physician. In the self-management dimension, elective physicians provide more precise and detailed explanations, highlighting their ability to foster better self-management. Differences in the communication dimension favour elective physicians at both factual and interpersonal levels, although there are no significant differences in patient’s ability to ask questions and receive answers. Across all dimensions, the time spent with patients emerges as a crucial factor influencing patient empowerment.

**Conclusions:**

The findings open up several avenues for further research, offering an initial understanding of the differences in patient empowerment between elective and contracted physicians. The results reveal that elective physicians are more likely to create a supportive environment for patient empowerment, underscoring the crucial role of physician-patient interactions in this process. However, given the limitations of the current methods, future research could explore these differences through alternative approaches, such as surveys or interviews, to provide a more comprehensive understanding.

**Supplementary Information:**

The online version contains supplementary material available at 10.1186/s12913-025-12635-7.

## Background

Patient empowerment and the doctor-patient relationship are closely related [1–3]. A positive relationship with the physician influences the treatment process [1, 4] and the ability to cope with one’s illness [1, 2]. Patients want to be treated as human beings by all healthcare professionals, not just physicians. This involves listening to patients, valuing their opinions, and acknowledging them as the experts of their own bodies. Linked to patient empowerment is supporting patients by the trust of the healthcare staff. Considering the relationships, patients benefit from individual information. Respecting the patient’s individual need for information reduces anxiety. It creates a sense of control, which has a positive effect on the development of the disease and its treatment. Patients’ perceived control over medical decisions and personal responsibility for their health and daily life are also important factors in patient empowerment [5].

Patient empowerment is a complex concept. Thus, the definitions of patient empowerment vary [6]. Funnell et al. define patient empowerment as an interactive process between healthcare professionals and patients, which aims to transfer knowledge and enhance patients’ ability to improve their quality of life [7]. Since then, it has been one of the most frequently cited definitions of patient empowerment [6, 8]. Acuña Mora et al. note that there are various definitions, and some authors partly cite more than a single definition in their publications [6]. We can classify definitions into different areas: Definitions may focus on the patient empowerment process or on principles (e.g. autonomy, self-management). Structuring definitions based on levels (patient level, healthcare provider level, healthcare system level) [8] lead to another classification. Other definitions focus on episodes like the doctor-patient interaction, implying that patient empowerment only occurs through a communicative process and a partnership relationship between health professionals and patients. The relationship should be egalitarian, equal, and based on information exchange and the transmission of knowledge, the acquisition of skills and abilities, and motivation [9].

## Theoretical framework

Compared to international standards, Austria’s system of elective and contracted physicians is unique [10]. In light of this context, the study emphasises the differences in patient empowerment between these two groups. Contracted physicians are doctors who have entered into a contract with a social health insurance provider, while elective physicians are those who do not have such a contract [11]. The proportion of elective practitioners in the outpatient healthcare sector is continuously increasing: in 2016, 55% of practitioner physicians were elective physicians. In comparison, ten years earlier, only 47% of physicians operated without a social health insurance contract [11]. This trend coincides with a 4% decrease in contracted physicians and a nearly 31% increase in elective physicians between 2012 and 2021 [12]. In this context, it is also worth noting that out-of-pocket payments in Austria’s outpatient sector constitute 19% of total out-of-pocket expenditures, below the OECD38 average of 22% [13]. Because of the insurance remunerations in Austria, contracted physicians try to generate many insurance-chargeable service positions, which means that the diagnostic, supportive, and motivating factors in the doctor-patient conversation are neglected, and little time is spent on the person [10]. On the other hand, patients value the individual approach and more time spent by the elective physicians. However, providing detailed information about diagnoses and further treatment is also important to patients who visit an elective doctor [14]. This study focuses on elective and contracted physicians in Vienna as a metropolitan area. Particularly in urban areas, the density of physicians of both kinds is high. Especially in the big cities, more and more elective physicians have settled since they can freely choose their place of practice, unlike contracted physicians [11, 15]. We expect that, because the contracted doctor caters to increase the service positions, empowerment of the patients will be lower, which leads to the following research question:

- RQ: *What differences in patient empowerment between elective and contracted physicians in internal medicine can be determined by analysing online comments?*

To consider patient empowerment in a structured way but still in various facets, we define dimensions with the following detailed literature review considering single quantitative and qualitative studies and review papers. Dimensions of patient empowerment are defined differently and reflect the respective focus of the study [3, 9, 16–20]. For example, in the study by Cerezo et al. the measurement tools of patient empowerment are examined and ten particular dimensions are derived [17]. The study by Fotoukian et al. has focused on the elderly population and derives dimensions that primarily address self-management as the most important factor for seniors [18]. In contrast, research by Moretta Tartaglione et al. addresses patient-centered healthcare and co-creation value, respectively, patient satisfaction [9]. Since the definition and comprehensive dimensions of Moretta Tartaglione et al. are strongly related to the interaction with healthcare professionals, these dimensions have been used as a basis for our further research:

- Health literacy: Dealing with health-related information in everyday life - obtaining, understanding and using information [3, 9, 17, 19, 20].
- Shared decision making: Shared decision making and the competence to make decisions [3, 9, 16–20].
- Self-management: The individual actions, behaviours, and coping strategies of the ill person [3, 9, 16–20].
- Communication between physicians and patients: Interactive and reciprocal communication, receiving information [3, 9, 16–19].

### Dimension health literacy

Health literacy moderates the doctor-patient relationship. When healthcare professionals consider patients’ health literacy in communication, the unmet need for information decreases and the perceived social support from physicians and nurses improves [21]. Special training of medical professionals in communication can promote health literacy and improve patients’ treatment outcomes, self-efficacy, and treatment adherence. Medical professionals must recognise patients’ communication needs [22] and use patient-centred communication [23]. Most importantly, communication should be done in simple language without medical terminology. Written and visual aids may be used [24].

- *H1: Elective physicians take patients’ health literacy into account in the conversation more than contracted physicians.*

### Dimension shared decision-making

Shared decision-making can be both a component and an outcome of patient empowerment [17]. The physician contributes the medical expertise and the patient the knowledge about the individual personal situation [25]. Above all, knowledge or adequate information transfer is essential to joint decision-making and linked to health literacy. Patients, therefore, require individualised care in doctor-patient contact [26]. However, shared decision-making does not only consist of conveying information. It is also necessary to engage in discussion, identify risks, answer questions, and determine the patient’s goals to make a joint decision [27].

- *H2a: Elective physicians provide more detailed information about treatment options compared to contracted physicians.*
- *H2b: Elective physicians are more likely to make treatment decisions collaboratively with their patients compared to contracted physicians.*

### Dimension self-management

Self-management can also be promoted through interaction with healthcare providers. Appropriate communication of the healthcare professionals, empathy, emotional support, and trust play a substantial role in the relationship between the two parties [28, 29]. Above all, the approach should be a person-centred, cooperative relationship, and holistic [30]. A literature review about self-management of patients with COPD and asthma found that patients particularly benefit from open communication with understandable language, the opportunity for self-assessment of their health status and the opportunity to ask questions [29]. If, besides the medical problems, the patient’s needs are addressed, and the everyday challenges of the patient are considered in the conversation, the doctor-patient relationship improves, and patients are more willing to implement and use activities and medications suggested by the medical staff. The doctor-patient relationship improves when a doctor addresses the medical issues, the patient’s needs, and everyday challenges during their conversation. As a result, patients are more likely to follow through with the activities and medications their medical team recommends. Furthermore, information transfer (explanations of diagnosis, prognosis, management, resources and advice) should also occur through two-way dialogue [31].

- *H3: Elective physicians offer more detailed explanations and demonstrate a greater level of knowledge compared to contracted physicians.*

### Dimension communication

Listening to, sharing and discussing information with the patient are the basis of patient-centred communication [32], which can also build a trusting relationship [33]. Considering the patient’s preferences, needs, and values and showing them empathy, compassion, and respect are important for such a relationship. The patient should be seen as an expert on their own health [32]. In addition, it is also important for patients to be able to express their concerns and fears in the conversation and for the medical professional to be honest [34]. Giving patients time to ask questions and respond accordingly and addressing medical problems are essential, too [35].

- *H4a: Elective physicians communicate in a more patient-centred manner than contracted physicians.*
- *H4b: Elective physicians provide more precise answers to questions than contracted physicians.*

### Barriers to patient empowerment

However, factors that influence implementing or promoting patient empowerment at the individual levels need to be considered. Structural challenges such as increased patient volume [36, 37], low structural resources [28, 36–38] or prevailing organisational policies [39], and high workload [28] harm the process. In addition, personal factors, such as a lack of physician training in patient communication [36, 37], are barriers to patient empowerment. The high time pressure or the lack of time resources are obstacles [28, 36–39]. The paternalistic relationship between patient and physician influences patient empowerment, too [40]. While the relationship between the two parties has changed over the past decades (i.e., based on the easier availability of health information), the paternalistic power relationship still exists [41]. Halvorsen et al. note that the physicians must initiate the patient empowerment process, and thus, the relationship retains the paternalistic character, and an adequate empowerment process can be hindered [40].

## Methods

### Quantitative content analysis

DocFinder.at is a search and review platform for practicing physicians in Austria’s outpatient sector. The platform lists physicians, their specialities, phone numbers, locations, opening hours, and the health insurance providers they accept. Patients share their experiences, provide feedback, and assist others in choosing a physician. Quality control measures are in place to filter out inappropriate content [42].

To collate broad data, a thematic quantitative content analysis of comments (see, e.g. Lockie et al. [43]) on DocFinder.at has been chosen as the method (selection unit: elective physician and contracted physician, analysis unit: comments on DocFinder.at). The evaluation platform DocFinder.at has been selected since the physicians on the other Austrian platforms (aerztekammer.at/arztsuche, arztsuche.netdoktor.at, arztsuche24.at) do not provide reviews at all or are rarely used. Thus, DocFinder.at is this study’s only relevant rating platform (primarily due to the method used). The possible alternative - ratings on Google.at - is also excluded, as it is impossible to distinguish between elective and contracted physicians. On DocFinder.at, all German comments from the selected physicians (see sample and inclusion/exclusion criteria) have been included in the analysis. In a further step, no relevant comments (no variable of patient empowerment codable) have been excluded from the sample, thus enabling an independent selection of the comments. By analysing the content of comments, we filtered relevant information and tested hypotheses using statistical tests with SPSS 26.

#### Codebook

The codebook consists of seven categories and 48 variables. An initial deductive approach informed the codebook. The reviewed literature on patient empowerment and the derived dimensions are the codebook’s basis. For all variables, a description and their characteristics with anchor examples (based on comments on DocFinder.at) have been developed to ensure the best possible assignment of the content of the comments [44, 45]. Text examples of the variables and their characteristics can be found in the Supplementary Material 1 (which is provided in German to maintain the original context). The written respective characteristics of the variables in the codebook are additionally presented quantitatively, which enables statistical calculations.

The first category is *formal variables*, which summarise general formal content. These formal contents included the assigned ID of the physician (to achieve anonymisation), the overall rating, the type of physician (elective or contracted physician), the gender of the physician, the comment number, the tendency of the comment (neutral, positive, negative), the time of writing, and the length of the comment.

The category *health* included two variables that provide information about the reason for the visit (preventive care, first visit, checkup, second opinion) and the health condition (chronic illness, acute illness). Since the reason and health condition are not observable in each comment, there was an additional option to code “No specification”.

The third category comprises two variables related to the information about the time spent by the physician (general and for questions) and constitutes the category *Time*. As shown in the literature, the factor of time is important for all dimensions of patient empowerment [28, 36–39] and thus the category *Time* is not assigned to any specific category of patient empowerment (category 4–7).

We generate four further categories in relation to the defined dimensions of patient empowerment with subsequent variables derived from the literature reviewed:

- Category: Health literacy
- Category: Shared decision making
- Category: Self-management
- Category: Communication

#### Sample

Several inclusion and exclusion criteria have been used for sampling. In order to obtain representative results, this study focuses on the reviews of internal medicine doctors. According to a study on German rating platforms by Emmert and McLennan, internists are the most frequently rated specialists [46] and with 4,492 physicians, internists represent the second largest group of specialist physicians in Austria after general practitioners [47]. Only internists (without considering the additional specialisation within the field) have been included to improve comparability. A study by McLennan on several rating platforms in Switzerland shows that written reviews in the form of comments have increased significantly since 2016 [48]. Due to the increase in the number and the growing importance of online rating platforms, comments from the last six years have been used for this study. The sample includes physicians who received comments within the past six years; although some of these physicians may have been practising in the outpatient sector for a more extended period or may have received comments prior to this timeframe, only the comments from the last six years were considered for the analysis. At least six written comments per physician are required to achieve a meaningful outcome [49]. An inclusion criterion was established, mandating at least nine comments per medical professional. The number of nine comments results from the testing of the codebook, where it was found that no variable relevant to patient empowerment could be coded for approximately 25–30% of the comments.

Regarding distinguishing between elective and contracted physicians, only elective physicians who had exclusively listed “Privat” or “Wahlarzt”/”Wahlärztin” (without additional social health insurance) have been analysed. In the case of contracted physicians, only physicians who had at least listed the Österreichische Gesundheitskasse (ÖGK) have been included in the analysis. Regionally, the analysis has been limited to physicians in Vienna. For each group (contracted vs. elective), comments from 20 randomly selected physicians were collated. This data collection totalled 1,153 comments for the thematic coding.

#### Reliability analysis

Two assessors have checked and tested the codebook independently. The first person has checked the consistency of the content and comprehensibility of the codebook without coding comments. The feedback is included in the codebook. Subsequently, another person tested the codebook based on 63 comments (two physicians), which have not been included in the survey. Based on the performed coding of the 63 comments by coder A (one author of the paper) and coder B (another person), intercoder reliability following Holsti [50] was computed, leading to the following coefficients indicating a high intercoder reliability [44] (Table 1).

**Table 1:** Reliability coefficient

Categorie	Reliability coefficient
Formal variables	0,99
Health	0,94
Time	0,96
Health literacy	0,74
Shared decision making	0,80
Self-management	0,89
Communication	0,85

A reliability coefficient of close to 1.0 should be achieved for formal categories and above 0.80 for content-related categories [44]. These thresholds are met for all categories except for health literacy. For the health literacy category, a comparison of coder A to coder B yields that coder B made fewer codes, reducing the number of matching codes. To increase reliability, the descriptions in the codebook and the coding rules have been revised again for this category. Few coding were made for the health literacy and shared decision making category.

#### Data evaluation and analysis

Of the 1,153 comments, 649 are attributed to elective physicians and 504 to contracted physicians. From 03.04.2022 to 09.04.2022 the comments were copied from DocFinder.at and saved. The thematic coding was performed using MAXQDA. After completing the coding, the data was exported to Excel, and the codes were converted into numbers according to the codebook. Through this process, the quantitative data could be imported into SPSS. Patient empowerment variables have been collapsed into index variables, meaning the higher the respective value, the more elements and signals of patient empowerment were observed. Using appropriate statistical tests, the differences or correlations (Mann-Whitney U Test for ordinal data comparisons, Pearson Chi-Square Test for categorical comparisons) and the effect size (Pearson correlation, Phi Coefficient) are determined. Because the factor time is relevant for all dimensions of patient empowerment, the correlation between the individual variables of the respective dimension and the variable time has been calculated with the Spearman Rank Correlation.

The significance level is set at *p* < .05. For *p*-values between 0.06 and 0.10, the results are seen as a trend. The effect size of all correlation coefficients was determined oriented according to the outline of Cohen (1992):

- *r* = .10 corresponds to a weak effect
- *r* = .30 corresponds to a medium effect
- *r* = .50 corresponds to a strong effect

## Results

### Sample characteristics and general differences

One thousand one hundred fifty-three comments have been coded, of which 504 comments relate to contracted physicians (43.7%) and 649 comments relate to elective physicians (56.3%). Only comments for which at least one variable of patient empowerment is observed are considered relevant, resulting in 912 comments for the final sample of this study (79.1%). On average, contracted physicians have 25 comments and elective physicians 32 comments. Two elective physicians are outliers with over 100 comments, whereas only one physician with over 100 comments is in the contracted physician group. For contracted physicians, an average of 18.6 and for elective physicians, an average of 27 relevant comments have been written over the past six years. The median shows that 14.5 relevant comments are written for elective physicians and 13 for contracted physicians, which means that there is only a minor difference. For further calculations, only the relevant comments (*n* = 912) and thus 372 comments from contracted physicians (40.8%) and 540 comments from elective physicians (59.2%) are used.

#### Comment length

The number of words assesses the length of the comments. Comments of contracted physicians (mean = 59, median = 50, mean rank = 458.35) are only minimally longer than the comments of elective physicians (mean = 57.74, median = 49, mean rank = 455.22) and therefore not significantly different according to a Mann-Whitney U Test (U = 99750, *p* = .860). In both groups outliers are identified, for elective physicians with 252 words and for contracted physicians 198 words.

#### Longitudinal timing of comments

Since the timestamp on DocFinder.at it is only given as “from a DocFinder user from x years ago”, there cannot be a detailed year assigned; only the period based on the time of the comment analysis is possible. The time of writing of all comments was, on average, 3.24 years ago (median = 4). Comments from contracted physicians were written on average 3.71 years ago (median = 4, mean rank = 517.34) and comments from elective physicians were written 2.91 years ago (median = 3, mean rank = 414.59). Looking into the relative distribution, most comments were written 5.0 years ago, and thus, from 04/2016 to 04/2017. A Mann-Whitney U Test reveals a significant difference (U = 77806.00, *p* < .001).

#### Quantitative rating of physicians

The average quantitative rating (out of a maximum of 5.0) for contracted physicians is 4.626 stars (median = 5, mean rank = 424.89) and 4.963 stars (median = 5, mean rank = 478.28) for elective physicians which are statistically different (U = 88681.5, *p* < .001).

#### Sentiment of comments

94.4% are positive and 4.8% are negative. Only 0.8% of all relevant comments are neutral. There is a slightly different relative sentiment distribution between the elective and contracted physicians. In the case of elective physicians, 98.7% of the comments are positive, compared to 88.2% of contracted physicians. This difference is significant according to a Fishers Exact test (*p* < .001).

#### Reason for consultation and health status

Only in a small proportion of comments do the patients describe the reason for consultation and/or their health status. More than 80% of comments do not include the reason for consultation or health status.

### Hypothesis testing

#### Time for patients

Comments have been coded with two different variables regarding time spent in consultation. One is time in general, and the other is related to questions. In the patients perception elective physicians spent significantly more time on patients (mean = 3.47, median = 3.00, mean rank = 202.69) in general (U = 10790.00, *p* < .001) as well as significantly (U = 93.00, *p* = .046) more time on patients’ questions (mean = 3.29, median = 3, mean rank = 19.57) compared to contracted physicians (time general: mean = 3.07, median = 3.00, mean rank = 148,24; time for questions: mean = 2.85, median = 3.00, mean rank = 14.15). The calculated effect size is rs = 0.253 for the variable time in general and rs = 0.343 for the variable time for questions, which corresponds to a medium effect in both cases.

#### Health literacy

For the presentation of the results of the health literacy dimension, we report the following sub-areas:

##### Relationship-relevant aspects

Drawing on the physician-patient relationship, we observe differences in the patient’s health literacy in the doctor-patient relationship (responding to patients, listening, asking questions, and doctor showing interest). A significant difference (U = 7265.00, *p* < .001) is found with a medium effect size (*r* = .204). We observed less relationship relevant aspects (mean = 2.91, median = 3.00, mean rank = 121.55) for contracted physicians compared to the elective physicians (mean = 3.37, median = 3.00, mean rank = 152.01). In addition, the Spearman Rank Correlation is used to test whether the relationship is related to the factor time. A highly significant medium positive correlation of these two variables was found (*r* = .373, *p* < .001).

##### Language aspects

We examined whether we find a difference between elective and contracted physicians and the expressions physicians use when talking to patients, which yield no significance according to Fisher’s Exact Test (*p* = .564), whether in the physician’s use of technical terms or simple language. Only a small number of reviews, however, indicated these language aspects. Thus, our data suggests that this is not very relevant to patients.

##### Communicative aspects

Finally, the communication by the physicians is investigated. We tested whether elective and contracted physicians could convey enough information by verbal or visual and in written information. The primary purpose of this section was to determine whether written or visual aids had been used and whether patients felt they received sufficient information. Only a few codes are found in the written and visual communication. Also, the highlighting of key points by physicians was mentioned only five times, which means that these variables have to be excluded from the presentation of results. In the area of communication, we further investigate whether patients subjectively felt that they received enough information. A medium and significant difference was found between the subjective feeling of having received enough information and the type of physician (φ = 0.248, *p* = .009). Reviews from contracted physicians showed a much lower frequency of providing information (11.3%) compared to elective physicians (18.9%).

#### Shared decision-making

We first investigate whether elective and contracted physicians differ regarding the presentation of treatment options. In total, in both cases, only slightly different treatment options have been reported in the reviews (*n* = 27). Contracted physicians have a slightly lower frequency (2.2%) than elective physicians (3.5%). With *p* = .322 (Fischer’s Exact Test), no significant difference is found between the presentation of treatment options and the type of physician. If treatment options had been discussed in principle in the conversation, how precisely the treatment options were discussed is relevant. This aspect is rarely described in the comments (*n* = 24), and subsequently, the Mann-Whitney U Test yields no significant difference between the two variables (U = 51.00, *p* = .819). However, when discussing treatment options associated with the time spent by physicians, a strong positive correlation of the two variables is observed (rs = 0.509, *p* = .037).

We observed discussing risks and answering questions about treatment options only three times, so these aspects of shared decision-making cannot be included in any further analysis of results. Similarly, joint decision-making between patients and physicians has been coded in only five comments.

#### Self-management

Self-management addresses two areas: knowledge transfer through explanations on the part of physicians and the relationship between physicians and patients.

We examine whether the two groups differ in knowledge transfer in terms of explanations, including specific explanations of test results, diagnoses, therapies, or general tips. A significant difference can be found with a *p*-value of *p* = .038 (U = 10378.00). Accordingly, elective physicians (mean = 3.51, median = 3.5, mean rank = 171.33) explain more accurately and understandably in conversation than contracted physicians (mean = 3.25, median = 3.00, mean rank = 150.59) do.

For the physician-patient relationship, specifically related to responsiveness to patients’ individual situations, no significant difference for the type of physician can be detected (*p* = .083), yet Fischer’s Exact Test yields a *p*-value less than 0.10, implying a trend. Reviews of elective physicians (7.2%) showed more discussion of the individual situations than contracted physicians (1.9%).

On the relationship level in general, a significant difference (U = 6332.00, *p* = .011) in the doctor-patient relationship is found (trust, partnership and consideration of the personal situation). In the reviews of contracted physicians, the relationship levels (mean = 0.96, median = 1.00, mean rank = 119.17) were lower than elective physicians (mean = 1.07, median = 1, mean rank = 131.40).

We further examined the correlation of self-management with time. Both the area of explanations (*p* < .001) and the conditions for self-management at the relationship level (*p* = .038) are significantly related to the factor time. The effect size between time and explanations is medium with rs = 0.306, and between time and relationship level is medium with rs = 0.211.

#### Communication

In the communication dimension of patient empowerment, we focus on how the physician behaves toward the patient. For example, whether the physician took the patient seriously and showed respect and honesty toward the patient (factual level). On the other hand, it is considered whether the physician has responded to the individual fears on the interpersonal level, listened and shown understanding. A Mann-Whitney U test reveals that elective (mean = 1.04, median = 1, mean rank = 39.41) and contracted physicians (mean = 0.76, median = 1, mean rank = 29.72) differed highly (U = 418.00, *p* < .001) in conversation at the factual level. The effect size was also *r* = .410, corresponding to a moderately strong effect. A significant difference (U = 3498.00, *p* = .016) is also observed at the interpersonal level. The effect size here is *r* = .175, corresponding to a weak effect. Reviews of contracted physicians (mean = 3.06, median = 3, mean rank = 85.08) showed less interpersonal communication than the elective physicians (mean = 3.47, median = 3.5, mean rank = 103.35).

As in the other dimensions, the factor time is associated with the variables. For both the factual (*p* = .015) and the interpersonal level (*p* < .001), a significant correlation to the variable time is found. Regarding effect strength, the factor time has a moderately strong to strong effect (rs = 0.441) on the factual level and a strong effect (rs = 0.509) on the interpersonal level.

In addition to the relationship variables, the possibility of asking questions in the conversation and the degree of answering the questions are investigated. There is no significant difference (*p* = .381) between the type of physician and the ability to ask questions. For the determination of significance, due to the expected cell frequency below 5 in two cells, Fischer’s Exact Test was used. No significant difference (*p* = .200) is found here either. Also, regarding answering questions, no significant difference (U = 1646.00, *p* = .404) between the two groups can be determined using the Mann-Whitney U Test.

## Discussion

Differences between the two cohorts have been demonstrated in three of four dimensions. Notably, time is a significant influencer in all dimensions. Elective physicians allocate significantly more time for patients, which is also a key factor in choosing them over contracted physicians [14]. In all dimensions, significant correlations are observed between the dimensions’ variables and the time factor, indicating that the amount of time physicians spend is central to patient empowerment, which is consistent with the literature reviewed [28, 36–39].

In the first dimension, health literacy, the study did not specifically assess patients’ health literacy regarding their ability to receive, understand, interpret, and apply information [39]. It focused on patients’ health literacy in the doctor-patient relationship and thus in the conversation between the two groups, with an emphasis on information transfer. In comparison with contracted physicians, elective physicians consider the relationship-relevant factors that are potentially conducive to the assessment of health literacy (responding to patients, listening, asking questions, and showing interest on the part of the physician) more than contracted physicians do. The form of expression used in verbal communication has an influence on the comprehension of information. According to Hersh et al., simple verbal language and avoiding medical terminology can promote patients’ health literacy by improving their understanding of the information [24]. In the context of the doctor’s language, whether using technical terminology or simple language, no significant correlation has been found. Neither elective nor contracted physicians promote patients’ health literacy through verbal expressions. Only a small number of 49 codes could be assigned to 912 comments in this area. However, the elective physicians succeed better in fulfilling the need for information in general, which is, according to Lubasch et al., an indication that the communication is adapted to the patients’ health literacy [21]. Indeed, it is not only adapted communication that better meets the patient’s information needs; there may also be other contributing factors.

We do not find a difference in shared decision-making. Elective and contracted physicians provide the same information about treatment options and discuss them in detail. In essence, it is necessary to recognise that this dimension posed methodological challenges in its evaluation, emphasising the need for further research to enable more precise conclusions regarding differences in shared decision-making due to the sometimes low number of codes.

In self-management, we observe significant differences between the two groups. According to Materese et al., patient knowledge is a central factor in self-management [51]. We find that elective physicians provide significantly clearer explanations compared to contracted physicians, which can improve patients’ knowledge and, thus, their self-management [52]. In the context of the physician-patient interaction, addressing the patient’s circumstances is also essential for improving self-management [31]. No significant results are found in this aspect of self-management. Despite that, if relevant relationship factors (trust, partnership, personal situation) are considered, elective physicians create a relationship level that is more conducive to self-management.

Communication adapted to the patient can increase patient engagement in physician contact and subsequently increase willingness to make decisions together [53]. Patient-centred communication can also transfer knowledge according to health literacy [22] and increase self-management through the generated knowledge [31]. Appropriate communication between physician and patient is a key factor in patient empowerment. In our study we see significant differences between the two groups at factual (taking patients seriously, respect, honesty) and interpersonal (addressing fears, listening, showing understanding) level, making elective physicians more conducive to patient empowerment on both levels of communication. Responding to patients’ questions is also part of effective communication [35]. With this regard, elective and contracted physicians do not differ. Both groups equally enable the asking of questions and provide also equally satisfactory responses. In summary, the relationship levels of communication and the option asking questions are essential influencing factors for patient empowerment and should be considered in every doctor-patient conversation.

### Limitations

Some limitations are worth noting. This research represents a one-sided perspective (patients’ perspective) of patient empowerment in the outpatient healthcare sector. Based on reviews, another possible limitation of this study is that usually, somewhat satisfied and rather dissatisfied consumers or patients write reviews, which might influence the findings as, in particular extreme cases, provide data. The results can only be seen as a tendency; a conclusion on the population of all physicians in the outpatient healthcare sector in Austria cannot be drawn. This is due on the one hand to the restriction of the specialty of internal medicine and on the other hand to the sample size of 20 physicians per group. In addition, only the metropolitan area of Vienna is examined, which means that the research results might only be extrapolated to other large cities in Austria but not all of Austria because of varying physician density. Notably is the unbalanced number of comments between the two groups which might overemphasise the performance of physicians with many comments.

From a methodological perspective, it is important to highlight that this study primarily focused on patient empowerment, thereby excluding an analysis of comment authenticity. Furthermore, this study did not assess the structure of the doctors’ profiles and whether they were paid or standard profiles. The influence of paid profiles is, therefore, not taken into account, although it is known that the characteristics of comments are influenced by paid profiles [54].

Regarding the codebook, it should be emphasised that its reliability is limited due to fewer codes in health literacy and shared decision-making. In addition, it has not been possible to code sufficiently for some areas or variables (e.g., shared decision making variable), so that no differences could be identified. This is because little or no information has been provided in the comments, and the method used is unable to generate meaningful results for individual areas. Therefore, it is important to start at this point and conduct further research using other methods like surveys or experiments.

As another limitation, it cannot be guaranteed that contracted physicians did not engage in elective practice before entering a contract and kept patients. This was notably observed when one contracted physician was explicitly identified as an elective physician in the written assessment. This physician was excluded from the sample. Nevertheless, it cannot be guaranteed that this circumstance is not also the case for other physicians in the sample as information is lacking.

Regarding the content of the comments, it is important to note that it is inherently challenging for patients to assess and evaluate the medical competence of physicians. Patients’ expectations may also influence this difficulty. Especially patients of elective physicians show a higher expectation towards elective physicians due to the additional costs [14]. Furthermore, specific individuals possess a greater capacity for reflective assessment of their interactions with physicians. Consequently, patients who are less able to reflect may tend to evaluate general conditions, such as waiting times in the waiting area, rather than their interactions with the physician. Moreover, it should be emphasised that patients incur additional costs for consulting an elective physician. Therefore, patients must have sufficient financial resources to consult an elective physician, and higher income is also usually related to a higher level of education. This leads to the assumption that patients of elective physicians might have a higher level of health literacy, enabling them to assess the contact with the physician better and formulate the evaluation on DocFinder.at accordingly.

To overcome the limitations of this study, several promising avenues for future research can be explored. One potential direction is to observe physician-patient interactions more closely, which could provide deeper insights into how these exchanges influence patient empowerment. Additionally, analysing the frequency of physician visits and healthcare utilisation could shed light on broader patterns in patient behaviour. Using focus groups and interviews would also be valuable, offering an opportunity to gather more in-depth, qualitative insights into the patient experience. Given the broad scope of patient empowerment, future studies may benefit from concentrating on specific aspects of empowerment, enabling a more focused and comprehensive examination.

As an additional focus, it would be helpful to incorporate general practitioners and orthopaedic doctors, among the most frequently reviewed specialities, into the research. This would provide a deeper understanding of how different specialities contribute to patient empowerment. Furthermore, while the study has focused primarily on urban areas, expanding to include decentralised regions would provide additional insights, particularly in areas with limited access to elective physicians. In these regions, analysing how physicians contribute to patient empowerment may reveal challenges different from those faced in metropolitan settings. Additionally, it would be valuable to explore how socio-economic and cultural factors influence empowerment, particularly for patients consulting elective physicians, who may have higher health literacy due to their socio-economic background. However, gathering such data through online platforms is challenging, and alternative methods such as surveys or interviews would be necessary to capture this important dimension.

Future research could incorporate data from other platforms, such as Google Reviews or local medical review sites, to better understand variations across cities and specialities, provided that their filtering and evaluation criteria align with those of DocFinder.at (which is not currently the case). This would facilitate a more robust comparison of patient empowerment across diverse regions and specialities. Furthermore, involving physicians in the study to understand their perspective on empowerment and the challenges they face in different practice contexts could offer a more balanced view and enrich the overall understanding of patient empowerment.

Moreover, expanding the research methodology to include quantitative surveys or experimental studies could significantly enhance the study’s depth.

## Conclusions

In health literacy, self-management, and communication, elective physicians seems to create more conducive conditions for patient empowerment. In this regard, the time resources of the physicians are of great importance. The individual subareas within these dimensions are related to the factor of time and show a positive effect on the individual aspects of patient empowerment. Elective physicians can create conditions conducive to patient empowerment, especially by having more time for the patients. Further research, including different methodologies, is necessary to examine the differences between elective and contracted physicians in various dimensions and confirm the results. This is the first study to examine the differences in patient empowerment between elective and contracted physicians and provides initial insight as a basis for further research.

## Supplementary Information

Supplementary Material 1.

## Data Availability

Data and materials can be requested from the corresponding author.
