# Supplementary material for: Differences of patient empowerment between elective and contracted physicians in internal medicine in Austria: a quantitative content analysis
Source: BMC Health Serv Res. 2025 Mar 31;25:476. doi: 10.1186/s12913-025-12635-7 (PMC11956238; doi:10.1186/s12913-025-12635-7)
Supplement: Supplementary file 1 — Supplementary Material 1. [file 12913_2025_12635_MOESM1_ESM.pdf]

CODEBUCH

PATIENT EMPOWERMENT –
Unterschiede zwischen
Wahl- und Kassenärzt:innen der
Inneren Medizin

Inhaltsverzeichnis

Inhaltsverzeichnis II
1 Einleitung 1
1.1 Allgemeine Codierregeln 1
1.2 Kategorienübersicht 2
2 Kategorie Formale Variablen 4
2.1 ID Ärzt:in 4
2.2 Gesamtbewertung 4
2.3 Arzttyp: Wahl/Kassen 5
2.4 Geschlecht Arzt/Ärztin 5
2.5 Kommentarnummer 5
2.6 Kommentarbewertung 6
2.7 Kommentarkategorie 6
2.8 Verfassungszeitpunkt 7
2.9 Länge des Kommentars 7
3 Kategorie Gesundheit 8
3.1 Angaben zum Konsultationsgrund 8
3.2 Angaben zum Gesundheitszustand 9
4 Kategorie Zeit 11
4.1 Zeit allgemein 11
4.2 Zeit für Fragen 12
5 Kategorie Gesundheitskompetenz 14
5.1 Eingehen auf Patient:innen 14
5.2 Nachfragen/Interesse zeigen 15
5.3 Zuhören des/der Arztes/Ärztin 15
5.4 Verwendung Fachsprache 16
5.5 Verwendung einfacher Sprache 17
5.6 Rückfragen Verständnis seitens Mediziner:in 17
5.7 Hervorheben von Eckpunkten 18
5.8 Verwendung schriftlicher Kommunikationsmittel 19
5.9 Verwendung visueller Kommunikationsmittel 19
5.10 Ausreichend Informationen 20
6 Kategorie Shared decision making 22
6.1 Aufzeigen von Behandlungsoptionen 22
6.2 Besprechung von Behandlungsoptionen 22
6.3 Erklärung von Behandlungsrisiken 23

6.4 Fragen zu Behandlungsoptionen ... 24
6.5 Gemeinsames Treffen der Entscheidung ... 25
6.6 Eingehen auf Ängste ... 26
6.7 Eingehen auf Patient:innen ... 26
7 Kategorie Selbstmanagement ... 27
7.1 Allgemeine Erklärungen ... 27
7.2 Erklärung Testergebnisse ... 28
7.3 Erklärung Diagnose ... 29
7.4 Erklärung Therapie ... 30
7.5 Tipps ... 31
7.6 Berücksichtigung der Bedürfnisse ... 32
7.7 Empathie ... 33
7.8 Vertrauen ... 34
7.9 Partnerschaftliches Verhältnis ... 34
7.10 Persönliche Situation ... 35
7.11 Meinung des/der Patient:in ... 36
7.12 Rückfragen Verständnis seitens Mediziner:in ... 36
8 Kategorie Kommunikation ... 37
8.1 Fragen stellen ... 37
8.2 Beantwortung der Fragen ... 37
8.3 Eingehen auf Ängste ... 38
8.4 Zuhören des/der Arztes/Ärztin ... 38
8.5 Zeigen von Verständnis ... 39
8.6 Zeigen von Respekt ... 39
8.7 Ehrlichkeit ... 40
8.8 Vertrauen ... 41
8.9 Ernst nehmen ... 41
9 Atmosphäre ... 42
10 Keine Angabe zu Patient Empowerment ... 43

1 Einleitung

Das Codebuch wurde als Messinstrument für die Forschungsarbeit mit dem Titel **Patient Empowerment - Unterschiede zwischen Wahl- und Kassenärzt:innen der Inneren Medizin** entwickelt.

Folgende Forschungsfrage soll im Zuge der Forschungsarbeit beantwortet werden:

Welche Unterschiede des Patient Empowerments zwischen Wahl- und Kassenärzt:innen der Inneren Medizin können durch die Analyse von Online-Kommentaren festgestellt werden?

Das Codebuch baut auf keinem bestehenden Codebuch auf und basiert auf den für die Forschungsarbeit definierten Dimensionen von Patient Empowerment: Gesundheitskompetenz, Shared decision making, Selbstmanagement und Kommunikation. Die aus der Literatur abgeleiteten wichtigen Aspekte in der Arzt-Patient-Beziehung zu den einzelnen Dimensionen wurden für die Erstellung der Variablen herangezogen.

Die für dieses Codebuch gültigen Auswahleinheiten stellen Wahl- und Kassenärzt:innen der Inneren Medizin auf DocFinder.at, die Analyseeinheiten die jeweiligen schriftlichen Bewertungen (Kommentare) zu den Mediziner:innen der Inneren Medizin dar.

1.1 Allgemeine Codierregeln

- Die Kommentare werden einzeln codiert. Je Kommentar sind alle Variablen codierbar. Ist eine Variable bei einem Kommentar nicht relevant, so wird diese Variable ausgelassen und nicht codiert. Ausgenommen sind formale Variablen und die Variablen zum Gesundheitszustand, diese müssen für jeden Kommentar codiert werden.

- Jede Variable verfügt über eine bestimmte Anzahl an Ausprägungen. Die Ausprägung, die zutrifft muss mit der jeweiligen Zahl codiert werden. Bei Unsicherheit der Zuordnung zu einer Ausprägung soll im Zweifelsfall die höherwertige Ausprägung codiert werden.
- Treffen für einen Satz oder Teil des Kommentars mehrere Variablen zu, werden immer alle Variablen mit der entsprechenden Ausprägung codiert.
- Jede Variable kann pro Kommentar nur einmal codiert werden. Wäre es möglich die Variable mehrfach mit unterschiedlichen Ausprägungen zu codieren, muss die höherwertige Ausprägung einmal codiert werden.
- Ist im Textteil des Kommentars keine relevante Variable für Patient Empowerment codierbar, so ist die Variable "v_pe_k" zu codieren. Die formalen Variablen werden dennoch codiert.

1.2 Kategorienübersicht

Das Codebuch besteht aus sieben Kategorien und 48 Variablen. Für alle Variablen wurde eine genaue Beschreibung der Variablen und der dazugehörigen Ausprägungen mit Ankerbeispielen (anhand von Kommentaren auf DocFinder.at) erstellt.

Die Kategorien gliedern sich wie folgt:

**Kategorie-Nr.**	**Kategorienname**
1	Formale Variablen
2	Gesundheit
3	Zeit
4	Gesundheitskompetenz
5	Shared decision making
6	Selbstmanagement
7	Kommunikation

In den folgenden Kapiteln werden die Kategorien genauer beschrieben. Dafür wird die Kategorie als gesamte Kategorie kurz beschrieben und anschließend die Variablen mit den jeweiligen Bezeichnungen, Definitionen, Ausprägungen und Ankerbeispielen genau erläutert.

2 Kategorie Formale Variablen

Die erste Kategorie stellt die Kategorie Formale Variablen dar, welche allgemeine formale Inhalte zusammenfasst. Zu diesen formalen Inhalten zählen die vergebene ID des/der Arztes/Ärztin, die quantitative Gesamtbewertung, der Arzttyp (Wahl- oder Kassenarzt/-ärztin), das Geschlecht des/der Arztes/Ärztin, die Kommentarnummer, die quantitative Kommentarbewertung, das Stimmungsbild des Kommentars (neutral, positiv, negativ), der Verfassungszeitpunkt und die Länge des Kommentars. Die formalen Variablen müssen für alle Kommentare codiert werden. Somit ist z. B. bei dem/der Arzt/Ärztin mit der ID 5, für jeden Kommentar die ID 5, sowie die jeweilige quantitative Gesamtbewertung, der Arzttyp und das Geschlecht anzugeben.

2.1 ID Ärzt:in

Um eine Anonymisierung der Ärzt:innen zu erreichen wird eine ID mittels einer ganzen Zahl vergeben.

<u>Variablenname:</u>
v_ID

<u>Codierung:</u>
Ganzzahl fortlaufend

2.2 Gesamtbewertung

Die Gesamtbewertung setzt sich aus der quantitativen Bewertung aller Kommentare, die für diese(n) Arzt/Ärztin vergeben wurden, zusammen und ist neben den allgemeinen Angaben auf DocFinder.at ersichtlich und für jeden Kommentar des/der Artes/Ärztin gleich. Die Erfassung der quantitativen Kommentarbewertung erfolgt unter der Variable v_kb.

<u>Variablenname:</u>
v_gb

Codierung:

Zahl (Punktewert mit einer Kommastelle durch Beistrich getrennt)

2.3 Arzttyp: Wahl/Kassen

Die Variable Arzttyp erfasst, ob der/die Arzt/Ärztin ein(e) Wahl- oder Kassenarzt/-ärztin ist.

Variablenname:

v_typ

Codierung:

0 = Kassenarzt/-ärztin

1 = Wahlarzt/-ärztin

2.4 Geschlecht Arzt/Ärztin

Diese Variable erfasst das Geschlecht des/der Arztes/Ärztin.

Variablenname:

v_geschlecht

Codierung:

1 = männlich

2 = weiblich

2.5 Kommentarnummer

Fortlaufende Nummer der Kommentare zu einem/einer Arzt/Ärztin.

Variablenname:

v_komment

Codierung:

Ganzzahl fortlaufend

2.6 Kommentarbewertung

Die vergebene quantitative Bewertung des Kommentars.

Variablenname:

v_kb

Codierung:

Zahl (Punktewert mit einer Kommastelle durch Beistrich getrennt)

2.7 Kommentarkategorie

Diese Variable erfasst das Stimmungsbild des Kommentars.

Variablenname:

v_kat

Codierung:

0 = negativ

1 = neutral

2 = positiv

Kommentarbeispiel	Codierung
Dr. … nimmt sich absolut keine Zeit, um auf irgendetwas einzugehen. Stellt nur am Anfang eine Frage, danach nichts mehr. Ich habe sogar dann selber nachgefragt, aber meine Fragen wurden eher abgelehnt, als beantwortet.<br><br>Meines Erachtens eine Frechheit, die Patient:innen wie am Fließband durchzuwinken.	Dieser Kommentar würde als **negativ** (0) codiert werden, da klar hervorgeht, dass nicht auf die Bedürfnisse/Fragen des/der Patient:in eingegangen wurde und klare negative Formulierungen enthalten sind.
Kurze Wartezeit und freundliches Team. Nach Untersuchungen Befunde schnell besprochen.	Dieser Kommentar würde als **neutral** (1) codiert werden, da keine positiven oder negativen Formulierungen enthalten sind. Neutral sollte immer nur dann codiert werden, wenn keine positive oder negative Tendenz erkennbar ist oder ein Teil positiv und ein Teil negativ formuliert wurde.

Von Beginn an - Aufruf - Vorbereitung, Untersuchung selbst und Nachbetreuung - verlief alles bestens. Ich wurde, weil mir nach der Untersuchung schwindlig war, sogar angezogen! Das ist nicht selbstverständlich - ein freundlicher Service. Ich komme sicher wieder auch für weitere Untersuchungen. Vielen Dank.	Dieser Kommentar würde als **positiv** (2) codiert werden, da hier deutlich hervorgeht, dass der/die Patient:in zufrieden war und zusätzlich anführt, bei Bedarf wieder vorstellig zu werden.

2.8 Verfassungszeitpunkt

Der Verfassungszeitpunkt bezieht sich auf den Zeitpunkt des Verfassens des Kommentars. Es wird nicht das Kalenderjahr angeführt, da dies aus DocFinder.at nicht klar ablesbar ist.

Variablenname:
v_zeitp

Codierung:
0 = Von einem DocFinder Nutzer vor x Monaten (unter 1 Jahr)
1 = Von einem DocFinder Nutzer vor einem Jahr
2 = Von einem DocFinder Nutzer vor 2 Jahren
3 = Von einem DocFinder Nutzer vor 3 Jahren
4 = Von einem DocFinder Nutzer vor 4 Jahren
5 = Von einem DocFinder Nutzer vor 5 Jahren
6 = Von einem DocFinder Nutzer vor 6 Jahren

2.9 Länge des Kommentars

Diese Variable erfasst wie viele Wörter ein Kommentar aufweist. Es wird die Anzahl der Wörter angegeben, alleinstehende Satzzeichen werden nicht als Wort gerechnet.

Variablenname:
v_laenge

Codierung:
Ganzzahl

3 Kategorie Gesundheit

Die Kategorie Gesundheit beinhaltet zwei Variablen, die die Angaben über den Konsultationsgrund des Besuchs und den Gesundheitszustand erfassen. Da nicht sichergestellt werden kann, dass immer der Grund und der Gesundheitszustand angeführt werden, gibt es bei beiden Variablen die Option „Keine Angaben" zu codieren. Wenn angeführt wurde, dass der/die Patient:in seit Jahren in Behandlung ist, erfolgt NICHT automatisch auch die Codierung der Variable v_zustand mit der Ausprägung 2 = chronische Erkrankung, selbiges gilt auch für die Variable v_konsul mit der Ausprägung 3 = Kontrolle. Für diese Angaben müsste 0 = keine Angaben codiert werden.

3.1 Angaben zum Konsultationsgrund

Diese Variable erhebt Angaben über den Konsultationsgrund auf den sich der Kommentar bezieht. Wenn keine Ausführungen zum Konsultationsgrund gemacht werden, dann ist 0 = keine Angaben zu codieren.

<u>Variablenname:</u>
v_konsul

<u>Codierung:</u>
0 = keine Angaben
1 = Vorsorge
2 = Erstvorstellung
3 = Kontrolle
4 = Zweitmeinung

**Beispiel**	**Codierung**
Kam zur Vorsorgeuntersuchung, …	Es wird die Ausprägung **Vorsorge** (1) codiert.<br>Diese Ausprägung wird gewählt, wenn die Vorsorge explizit erwähnt wird.

Ich war zum ersten Mal bei Dr. …	Es wird die Ausprägung **Erstvorstellung** (2) codiert.<br>Diese Ausprägung wird gewählt, wenn angegeben wird, dass es sich um eine erstmalige Vorstellung bei diesem/dieser Arzt/Ärztin handelt.
Ich und meine Frau waren nach einer Covid Infektion zur Kontrolle…	Es wird die Ausprägung **Kontrolle** (3) codiert.<br>Unter dieser Ausprägung fallen alle Kommentare bei denen explizit Kontrolle angeführt wird. Nicht codiert werden hier Angaben über die Behandlung über Jahre hinweg, diese werden unter 0 = keine Angaben erfasst.
...wollte ich eine 2. Meinung einholen.	Es wird die Ausprägung **Zweitmeinung** (4) codiert.<br>Diese Ausprägung wird gewählt, wenn die Zweitmeinung explizit erwähnt wird.

3.2 Angaben zum Gesundheitszustand

Diese Variable erfasst Angaben zum Gesundheitszustand. Nicht codiert wird diese Variable, wenn der/die Patient:in angibt, dass er/sie seit längerer Zeit in Behandlung ist aber keine Angaben über eine Erkrankung gemacht werden (es ist nicht automatisch davon auszugehen, dass der/die Patient:in eine chronische Erkrankung hat). Wenn keine Ausführungen zu einer akuten oder chronischen Erkrankung gemacht werden, dann ist 0 = keine Angaben zu codieren.

Variablenname:
v_zustand

Codierung:
0 = keine Angaben
1 = akute Erkrankung
2 = chronische Erkrankung

**Beispiel**	**Codierung**
Rasches und sehr kompetentes Reagieren auf akutes Problem.	Es wird die Ausprägung **akute Erkrankung** (1) codiert.<br>Diese Ausprägung wird gewählt, wenn Angaben zu einer akuten Erkrankung gemacht werden.

Ich bin durch meinen Diabetes sehr wählerisch…	Es wird die Ausprägung **chronische Erkrankung** (2) codiert.<br>Diese Ausprägung wird gewählt, wenn Angaben zu einer chronischen Erkrankung gemacht werden. Nicht codiert wird diese Ausprägung, wenn beschrieben wird, dass der/die Patient:in seit mehreren Jahren in Betreuung ist, dies wird unter 0 = keine Angaben erfasst.

4 Kategorie Zeit

Die dritte Kategorie besteht aus zwei Variablen bezüglich Angaben über die von dem/der Arzt/Ärztin aufgebrachte Zeit (allgemein und für Fragen) und stellen die Kategorie Zeit dar.

4.1 Zeit allgemein

Diese Variable beinhaltet die allgemeinen Angaben zur Zeit. Nicht beinhaltet ist Zeit für Fragen (v_zeit_f).

<u>Variablenname:</u>
v_zeit_a

<u>Codierung:</u>
1 = hat sich keine Zeit genommen
2 = hat sich wenig Zeit genommen
3 = hat sich ausreichend Zeit genommen
4 = hat sich viel Zeit genommen

**Beispiel**	**Codierung**
Gefühl, schnell abgehandelt zu werden.<br>… hat sich überhaupt keine Zeit genommen.	Es wird die Ausprägung **hat sich keine Zeit genommen** (1) codiert.<br>Diese Codierung erfolgt, wenn sich der/die Arzt/Ärztin keine/nicht/keineswegs Zeit genommen hat.
... nimmt sich wenig Zeit.	Es wird die Ausprägung **hat sich wenig Zeit genommen** (2) codiert.<br>Alle Kommentare, die zusätzliche Angaben mit negativer Tendenz im Vergleich zu "hat sich ausreichend Zeit genommen" aufweisen, werden hier zugordnet.
...nahm sich Zeit für mich.<br>...hat sich genügend Zeit genommen.	Es wird die Ausprägung **hat sich ausreichend Zeit genommen** (3) codiert.<br>Wenn sich der/die Arzt/Ärztin Zeit genommen hat ohne zusätzliche positive oder negative Tendenz, wird diese Ausprägung codiert.

Arzt/Ärztin, der/die sich viel Zeit genommen hat.<br><br>…nimmt sich wirklich Zeit.	Es wird die Ausprägung **hat sich viel Zeit genommen** (4) codiert.<br><br>Alle Kommentare, die zusätzliche Angaben mit positiver Tendenz im Vergleich zu "hat sich ausreichend Zeit genommen" aufweisen, werden hier zugordnet.

4.2 Zeit für Fragen

Diese Variable beinhaltet nur Angaben zur Zeit für Fragen. Nicht beinhaltet sind allgemeine Angaben zur Zeit (v_zeit_a).

<u>Variablenname:</u>
v_zeit_f

<u>Codierung:</u>
1 = hat sich keine Zeit genommen
2 = hat sich wenig Zeit genommen
3 = hat sich ausreichend Zeit genommen
4 = hat sich viel Zeit genommen

**Beispiel**	**Codierung**
…nimmt sich nicht mal Zeit für meine Anliegen.	Es wird die Ausprägung **hat sich keine Zeit für Fragen genommen** (1) codiert.<br><br>Diese Codierung erfolgt, wenn sich der/die Arzt/Ärztin keine/nicht/keineswegs Zeit für Fragen genommen hat.
…hat sich kaum Zeit für die Beantwortung meiner Fragen genommen.	Es wird die Ausprägung **hat sich wenig Zeit für Fragen genommen** (2) codiert.<br><br>Alle Kommentare, die zusätzliche Angaben mit negativer Tendenz im Vergleich zu "hat sich ausreichend Zeit für Fragen genommen" aufweisen, werden hier zugordnet.
…genug Zeit für meine Fragen und noch so kleinen Bedenken, die ich äußerte.	Es wird die Ausprägung **hat sich ausreichend Zeit für Fragen genommen** (3) codiert.<br><br>Wenn sich der/die Arzt/Ärztin Zeit für Fragen genommen hat ohne zusätzliche positive oder negative Tendenz, wird diese Ausprägung codiert.

…Arzt/Ärztin, der/die sich viel Zeit für Fragen genommen hat.<br>...nimmt sich Zeit für ein ausführliches Gespräch.	Es wird die Ausprägung **hat sich viel Zeit für Fragen genommen** (4) codiert.<br>Alle Kommentare, die zusätzliche Angaben mit positiver Tendenz im Vergleich zu "hat sich ausreichend Zeit für Fragen genommen" aufweisen, werden hier zugordnet.

5 Kategorie Gesundheitskompetenz

Die einzelnen Variablen beziehen sich auf relevante Aspekte zur Gesundheitskompetenz in der Arzt-Patient-Beziehung und wurden aus der Literatur abgeleitet.

5.1 Eingehen auf Patient:innen

Diese Variable beinhaltet, ob der/die Patient:in das Gefühl hatte von dem/der Arzt/Ärztin wahrgenommen worden zu sein und dass auf sie/ihn individuell eingegangen wurde. Nicht beinhaltet ist hier das aktive Nachfragen (v_bez_nf) oder Zuhören (v_bez_zu), hier ist das allgemeine Empfinden und nicht die Spezifizierung gemeint. Zusätzliche Signalwörter: Arzt/Ärztin ist engagiert.

<u>Variablenname:</u>
v_bez_e

<u>Codierung:</u>
1 = gar nicht
2 = eher weniger
3 = eher
4 = sehr

**Beispiel**	**Codierung**
...ist aber auf meine Beschwerden überhaupt nicht eingegangen.<br>...aber den/die Arzt/Ärztin hat das alles null interessiert.	Es wird die Ausprägung **gar nicht** (1) codiert.<br>Der/die Arzt/Ärztin ist nicht auf den/die Patient:in eingegangen.
…meine Schilderungen haben den/die Arzt/Ärztin nicht wirklich interessiert.	Es wird die Ausprägung **eher weniger** (2) codiert.<br>Der/die Arzt/Ärztin ist unzureichend auf den/die Patient:in eingegangen.
...geht auf den Patient:innen ein.	Es wird die Ausprägung **eher** (3) codiert.<br>Der/die Arzt/Ärztin ist auf den/die Patient:in eingegangen, ohne positive oder negative Tendenz.
...sieht die Patient:innen als Individuen und geht auf einen wirklich ein. Man fühlt sich von ihm/ihr bestens betreut und behandelt.	Es wird die Ausprägung **sehr** (4) codiert.<br>Der/die Arzt/Ärztin geht auf seine/ihre Patient:innen ein mit zusätzlich positiven Angaben.

5.2 Nachfragen/Interesse zeigen

Diese Variable beinhaltet das aktive Nachfragen/Interesse zeigen seitens des/der Arzt/Ärztin. Nicht beinhaltet ist hier das Zuhören (v_bez_zu) oder das allgemeine Eingehen (v_bez_e) auf den/die Patient:in.

Variablenname:

v_bez_nf

Codierung:

1 = gar nicht

2 = eher weniger

3 = eher

4 = sehr

Beispiel	Codierung
…ist aber auf meine Beschwerden überhaupt nicht eingegangen.	Es wird die Ausprägung **gar nicht** (1) codiert.<br>Der/die Arzt/Ärztin hat im Gespräch keinerlei Interesse gezeigt oder nachgefragt.
Ich hatte das Gefühl, dass es ihn/sie nicht wirklich interessiert, was ich erzählt habe.	Es wird die Ausprägung **eher weniger** (2) codiert.<br>Der/die Arzt/Ärztin hat im Gespräch nur sehr wenig Interesse gezeigt oder nachgefragt.
…hat mir gezielte Fragen gestellt.	Es wird die Ausprägung **eher** (3) codiert.<br>Der/die Arzt/Ärztin hat im Gespräch Interesse gezeigt oder nachgefragt.
...ist sehr interessiert.<br>...und hat auch viele Fragen gestellt, um einiges abzuklären.	Es wird die Ausprägung **sehr** (4) codiert.<br>Der/die Arzt/Ärztin hat aktiv nachgefragt oder Interesse gezeigt.

5.3 Zuhören des/der Arztes/Ärztin

Diese Variable bezieht sich auf das aktive Zuhören seitens des/der Arztes/Ärztin. Nicht beinhaltet ist hier das aktive Nachfragen (v_bez_nf) oder das allgemeine Eingehen (v_bez_e) auf den/die Patient:in.

Variablenname:

v_bez_zu

Codierung:

1 = gar nicht

2 = eher weniger

3 = eher

4 = sehr

Beispiel	Codierung
…zuhören und nicht gleich irgendein Medikament verschreiben,…<br>…hörte mir überhaupt nicht zu.	Es wird die Ausprägung **gar nicht** (1) codiert.<br>Der/die Arzt/Ärztin hat nicht zugehört.
…habe nachgefragt, aber da ist er/sie mir nur ausgewichen.	Es wird die Ausprägung **eher weniger** (2) codiert.<br>Der/die Arzt/Ärztin hat so gut wie gar nicht zugehört.
Arzt/Ärztin, der/die einem zuhört.	Es wird die Ausprägung **eher** (3) codiert.<br>Der/die Arzt/Ärztin hat zugehört - keine zusätzlichen positiven/negativen Angaben.
...hat immer ein offenes Ohr.<br>…hat genau zugehört.	Es wird die Ausprägung **sehr** (4) codiert.<br>Der/die Arzt/Ärztin hat sehr aufmerksam zugehört - zusätzliche positive Angaben.

5.4 Verwendung Fachsprache

Diese Variable bezieht sich explizit darauf, ob medizinische Fachbegriffe im Gespräch verwendet wurden. Die Variable setzt den Fokus auf das Nicht-Verwenden von medizinischen Fachbegriffen.

Nicht beinhaltet ist das Verständnis von Erklärungen allgemein (v_erkl_allg), zu Testergebnissen (v_erkl_test), Diagnose (v_erkl_diag), Therapiemöglichkeiten (v_erkl_therapie) oder allgemein eine verständliche Sprache (v_sprach_einfach).

Variablenname:

v_sprach_fach

Codierung:

0 = Nein

1 = Ja

Beispiel	Codierung
…verwendete viele medizinische Wörter, die ich nicht verstanden habe.	Es wird die Ausprägung **Nein** (0) codiert. Nein wird codiert, wenn medizinische Fachbegriffe im Gespräch verwendet wurden.
…erklärt so, dass auch Nicht-Medizin Absolvent:innen diese verstehen können.	Es wird die Ausprägung **Ja** (1) codiert. Erklärungen und Gespräch verlief, ohne zu viele medizinische Fachbegriffe zu verwenden.

5.5 Verwendung einfacher Sprache

Diese Variable bezieht sich speziell auf die Ausdrucksweise des/der Arztes/Ärztin. Nicht beinhaltet ist hier das Verwenden von medizinischer Fachsprache/Fachwörter (v_sprach_fach).

Variablenname:
v_sprach_einfach

Codierung:
0 = Nein
1 = Ja

Beispiel	Codierung
… ich konnte dem/der Arzt/Ärztin im Gespräch leider nicht folgen.	Es wird die Ausprägung **Nein** (0) codiert. Die Sprache des/der Arztes/Ärztin war schwierig zu verstehen, sehr verwirrend, kompliziert und somit nicht einfach verständlich.
...wurde mir mit verständlichen Worten erklärt.	Es wird die Ausprägung **Ja** (1) codiert. Die Ausdrucksweise war einfach und leicht zu verstehen. Bezug wird zur sprachlichen Ausdrucksweise hergestellt.

5.6 Rückfragen Verständnis seitens Mediziner:in

Diese Variable beinhaltet explizit das Rückfragen seitens des/der Arztes/Ärztin, ob die Erklärungen/Angaben zur Diagnose, Therapie, Testergebnissen usw. verstanden wurden.

Variablenname:

v_kom_rück

Codierung:

0 = Nein

1 = Ja

Beispiel	Codierung
…es wurde nicht einmal gefragt, ob ich die Erklärungen überhaupt verstanden habe.	Es wird die Ausprägung **Nein** (0) codiert.<br>Der/die Arzt/Ärztin hat nicht nachgefragt, ob Erklärungen verstanden wurden.
Ich wurde gefragt, ob ich alles verstanden habe und noch Fragen habe.	Es wird die Ausprägung **Ja** (1) codiert.<br>Der/die Arzt/Ärztin hat nachgefragt, ob Erklärungen verstanden wurden.

5.7 Hervorheben von Eckpunkten

Diese Variable beinhaltet, ob der/die Arzt/Ärztin die wichtigsten Eckpunkte/Inhalte über Untersuchung(sergebnisse)/Diagnose/Therapie hervorgehoben hat. Nicht beinhaltet sind das Verständnis von Erklärungen allgemein (v_erkl_allg), zu Testergebnissen (v_erkl_test), Diagnose (v_erkl_diag), Therapiemöglichkeiten (v_erkl_therapie). Der Fokus liegt auf dem Hervorheben von wichtigen Informationen.

Variablenname:

v_kom_eck

Codierung:

0 = Nein

1 = Ja

Beispiel	Codierung
…die Erklärungen waren sehr verwirrend und ich wusste schlussendlich nicht, was genau jetzt wichtig war.	Es wird die Ausprägung **Nein** (0) codiert.<br>Die wichtigsten Erkenntnisse/Inhalte/Informationen wurden nicht hervorgehoben/betont.

Der/die Arzt/Ärztin hat die wichtigsten Untersuchungsergebnisse auf den Punkt gebracht und gut verständlich erklärt.	Es wird die Ausprägung **Ja** (1) codiert.<br>Die wichtigsten Erkenntnisse/Inhalte/Informationen wurden hervorgehoben/betont.

5.8 Verwendung schriftlicher Kommunikationsmittel

Diese Variable beinhaltet, ob der/die Arzt/Ärztin im Gespräch schriftliche Mittel zur Erklärung verwendet hat. Zu schriftlichen Mitteln zählen auch Aufklärungsbögen/Informationsmaterial und ob diese zum richtigen Zeitpunkt eingesetzt wurden.

<u>Variablenname:</u>
v_kom_schrift

<u>Codierung:</u>
0 = Nein
1 = Ja

**Beispiel**	**Codierung**
…der Aufklärungsbogen wurde nicht mal wirklich mit mir durchbesprochen, ich musste einfach nur unterschreiben.	Es wird die Ausprägung **Nein** (0) codiert.<br>Schriftliche Kommunikationsmittel (z. B. Aufklärungsbogen, Informationsmaterial, Zusammenfassungen über Therapie, Infos und Tipps für Alltag) wurde nicht im Gespräch verwendet.
…auch der Aufklärungsbogen wurde sehr ausführlich mit mir durchbesprochen.	Es wird die Ausprägung **Ja** (1) codiert.<br>Schriftliche Kommunikationsmittel (z. B. Aufklärungsbogen, Informationsmaterial, Zusammenfassungen über Therapie, Infos und Tipps für Alltag) wurde im Gespräch verwendet.

5.9 Verwendung visueller Kommunikationsmittel

Diese Variable beinhaltet, ob der/die Arzt/Ärztin im Gespräch visuelle Mittel zur Erklärung verwendet hat. Zu visuellen Mitteln zählen grafische Darstellungen, Modelle, Videos und ob diese zum richtigen Zeitpunkt eingesetzt wurden.

<u>Variablenname:</u>
v_kom_visu

Codierung:

0 = Nein

1 = Ja

Beispiel	Codierung
...es hätte mir auch geholfen, zu sehen, wie genau die Therapie angewendet wird.	Es wird die Ausprägung **Nein** (0) codiert.<br>Visuelle Kommunikationsmittel (z. B. Grafiken, Modelle, Videos) wurden nicht im Gespräch eingebaut.
…durch die Erklärung mit einer Grafik konnte ich mir auch besser vorstellen, wo genau die Ursache meines Problems liegt.	Es wird die Ausprägung **Ja** (1) codiert.<br>Visuelle Kommunikationsmittel (z. B. Grafiken, Modelle, Videos) wurden im Gespräch eingebaut.

5.10 Ausreichend Informationen

Bei dieser Variable geht es explizit um den Informationsgehalt, den der/die Arzt/Ärztin vermittelt, inklusive schriftliche Informationsmaterialien. Darunter fällt, ob der/die Patient:in das Gefühl hatten ausreichend Informationen erhalten zu haben. Auch Angaben zur Beratung werden hier codiert. Nicht beinhaltet sind das Verständnis von Erklärungen allgemein (v_erkl_allg), zu Testergebnissen (v_erkl_test), Diagnose (v_erkl_diag), Therapiemöglichkeiten (v_erkl_therapie).

Variablenname:

v_kom_visu

Codierung:

0 = Nein

1 = Ja

Beispiel	Codierung
Lediglich die Tatsache, dass mein nächste Kontrolltermin erst in 6 Monaten vereinbart wurde, deutet für mich darauf hin, dass alles ok war.<br>…die Info bleibt auf der Strecke: Vieles wird einfach nicht ausgesprochen.	Es wird die Ausprägung **Nein** (0) codiert.<br>Es wurden zu wenig oder keine Informationen an den/die Patient:in weitergegeben. Patient:in wurde nicht ausreichend informiert.
…gibt viele hilfreiche Informationen.<br>…sehr ausführliche Beratung.	Es wird die Ausprägung **Ja** (1) codiert.

	Es wurden ausreichend Informationen an den/die Patient:in weitergeben. Patient:in hatte das Gefühl ausreichend Informationen erhalten zu haben.

6 Kategorie Shared decision making

Die einzelnen Variablen beziehen sich auf relevante Aspekte zur gemeinsamen Entscheidungsfindung in der Arzt-Patient-Beziehung und wurden aus der Literatur abgeleitet.

6.1 Aufzeigen von Behandlungsoptionen

Diese Variable beinhaltet, ob grundsätzlich Alternativen/Behandlungsmöglichkeiten vorgeschlagen wurden. Nicht beinhaltet sind das Gespräch (v_bo_bespr), Fragen (v_bo_f) und Risiken (v_bo_risk) über Behandlungsoptionen. Es geht explizit darum, ob grundsätzlich überhaupt verschiedene Möglichkeiten ins Gespräch gebracht wurden.

Variablenname:
v_bo_aufzeig

Codierung:
0 = Nein
1 = Ja

**Beispiel**	**Codierung**
…keine Alternativen erwähnt.	Es wird die Ausprägung **Nein** (0) codiert.<br>Es wurden keine Behandlungsoptionen/Alternativen/Therapiemöglichkeiten vorgeschlagen.
...hat mir auch Alternativen aufgezeigt.	Es wird die Ausprägung **Ja** (1) codiert.<br>Es wurden verschiedene Behandlungsoptionen/Alternativen/Therapiemöglichkeiten vorgeschlagen.

6.2 Besprechung von Behandlungsoptionen

Diese Variable beinhaltet konkret das Gespräch über verschiedene Therapiemöglichkeiten. Fragen über Behandlungsoptionen beantworten (v_bo_f) und das Eingehen auf Risiken (v_bo_risk) der Behandlungsoptionen werden hier nicht berücksichtigt.

Variablenname:

v_bo_bespr

Codierung:

1 = gar nicht

2 = eher weniger

3 = eher

4 = sehr

Beispiel	Codierung
…ich hätte gerne Alternativen vorgeschlagen bekommen.	Es wird die Ausprägung **gar nicht** (1) codiert.<br>Behandlungsoptionen wurden nicht aufgezeigt und auch kein Gespräch darüber geführt.
…Therapievorschläge sind 08/15.	Es wird die Ausprägung **eher weniger** (2) codiert.<br>Behandlungsoptionen wurden erwähnt allerdings nicht darauf eingegangen.
...es wurden Alternativen erwähnt allerdings wurden mir diese nicht sehr ausführlich erklärt.	Es wird die Ausprägung **eher** (3) codiert.<br>Es wurden Behandlungsoptionen aufgezeigt, allerdings fehlen ausreichend Informationen.
...Beratung und Informationen über Behandlungsmöglichkeiten war sehr ausführlich.	Es wird die Ausprägung **sehr** (4) codiert.<br>Ein ausführliches Gespräch über Behandlungsoptionen hat stattgefunden und genügend Informationen wurden vermittelt.

6.3 Erklärung von Behandlungsrisiken

Diese Variablen bezieht sich explizit auf die Erklärung der Risiken von Behandlungsoptionen. Nicht beinhaltet sind Erklärungen zur Diagnose (v_erkl_diag), Therapie (v_erkl_therapie), Testergebnisse (v_erkl_test) oder allgemeine Erklärungen (v_erkl_allg).

Variablenname:

v_bo_risk

Codierung:

1 = gar nicht

2 = eher weniger

3 = eher

4 = sehr

Beispiel	Codierung
…die Risiken wurden allerdings nicht erklärt.	Es wird die Ausprägung **gar nicht** (1) codiert.<br>Risiken wurden nicht erwähnt/erklärt.
…ich hätte mir noch mehr Informationen zu den Gefahren der Behandlung gewünscht.	Es wird die Ausprägung **eher weniger** (2) codiert.<br>Risiken wurden grundsätzlich angesprochen aber nicht ausreichend erklärt.
Die Behandlung wurde mir mit den Risiken verständlich erklärt.	Es wird die Ausprägung **eher** (3) codiert.<br>Die Risiken wurden erklärt ohne positive oder negative Tendenz.
Sogar für die Erklärung der Risiken hat sich der/die Arzt/Ärztin sehr viel Zeit genommen und super erklärt.	Es wird die Ausprägung **sehr** (4) codiert.<br>Risiken wurden angesprochen und dem/der Patient:in ausreichend und verständlich erklärt.

6.4 Fragen zu Behandlungsoptionen

Diese Variable beinhaltet ausschließlich Fragen zu Behandlungsoptionen. Allgemeine Angaben zur Beantwortung von Fragen (v_f_beant) sind hier nicht gemeint.

Variablenname:

v_bo_f

Codierung:

1 = gar nicht

2 = eher weniger

3 = eher

4 = sehr

**Beispiel**	**Codierung**
Meine Fragen zu den verschiedenen Möglichkeiten wurden nicht gehört.	Es wird die Ausprägung **gar nicht** (1) codiert.<br>Der/die Arzt/Ärztin hat keine/nicht/keineswegs die Fragen zu Behandlungsoptionen beantwortet.
Auch bei mehrmaligen Nachfragen, bekam ich keine ausführlichen Antworten zu den Alternativen.	Es wird die Ausprägung **eher weniger** (2) codiert.<br>Fragen zu Behandlungsoptionen sind zwar beantwortet worden, allerdings nicht zur Zufriedenheit oder ausreichend.
Die Fragen zu den verschiedenen Behandlungen wurden mir alle beantwortet.	Es wird die Ausprägung **eher** (3) codiert.<br>Fragen zu Behandlungsoptionen wurden beantwortet ohne positive oder negative Tendenz.
Für meine vielen vielen Fragen zu den verschiedenen Möglichkeiten hat sich der/die Arzt/Ärztin sehr viel Zeit genommen und verständlich beantwortet.	Es wird die Ausprägung **sehr** (4) codiert.<br>Fragen zu Behandlungsoptionen wurden beantwortet, mit zusätzlich positiven Angaben (z. B. äußerst ausführlich, sehr genau, sehr umfassend).

6.5 Gemeinsames Treffen der Entscheidung

Diese Variable beinhaltet, ob der/die Arzt/Ärztin und der/die Patient:in die Entscheidung für/gegen eine Behandlung gemeinsam getroffen haben.

Variablenname:

v_ent

Codierung:

0 = Nein

1 = Ja

**Beispiel**	**Codierung**
Ich hatte nicht die Möglichkeit meine Entscheidung gemeinsam mit dem/der Arzt/Ärztin zu treffen.	Es wird die Ausprägung **Nein** (0) codiert.<br>Die Entscheidung für/gegen eine Behandlung wurde nicht gemeinsam getroffen.
In gemeinsamer Abstimmung haben wir eine Behandlungsoption ausgewählt.	Es wird die Ausprägung **Ja** (1) codiert.<br>Die Entscheidung für/gegen eine Behandlung wurde gemeinsam getroffen.

6.6 Eingehen auf Ängste

Diese Variable beinhaltet, ob der/die Ärztin auf Sorgen/Ängste eingegangen ist. Nicht beinhaltet sind hier Anliegen/Interessen/Wünsche - diese sind unter der Variable v_bed_e zu codieren.

Variablenname:
v_bez_angst

Codierung:
1 = gar nicht
2 = eher weniger
3 = eher
4 = sehr

Beispiel	Codierung
…ich konnte meine Angst vor der Behandlung nicht einmal dem Arzt/Ärztin mitteilen.	Es wird die Ausprägung **gar nicht** (1) codiert.<br>Die Sorgen/Ängste wurden im Gespräch nicht berücksichtigt.
Meine Angst spielte der/die Arzt/Ärztin herab.	Es wird die Ausprägung **eher weniger** (2) codiert.<br>Die Sorgen/Ängste wurden im Gespräch kaum berücksichtigt/nicht darauf eingegangen.
...versucht Sorgen und Ängste (z.B. vor Operationen) zu nehmen.	Es wird die Ausprägung **eher** (3) codiert.<br>Der/die Arzt/Ärztin nimmt die Sorgen/Ängste wahr und geht auf diese ein.
Meine Angst vor der Behandlung konnte mir von dem/der Arzt/Ärztin vollkommen genommen werden.	Es wird die Ausprägung **sehr** (4) codiert.<br>Die Sorgen/Ängste konnten dem/der Patient:in durch den/die Arzt/Ärztin genommen werden.

6.7 Eingehen auf Patient:innen

Diese Variable ist bereits in der Kategorie Gesundheitskompetenz angeführt, ist allerdings ebenfalls für die Kategorie Shared decision making von Bedeutung, wodurch die Variable hier aufgelistet wird. Die genaue Beschreibung erfolgt allerdings unter Punkt 5.1. Die Codierung wird nur einmal vorgenommen.

7 Kategorie Selbstmanagement

Die einzelnen Variablen beziehen sich auf relevante Aspekte zu Selbstmanagement in der Arzt-Patient-Beziehung und wurden aus der Literatur abgeleitet.

7.1 Allgemeine Erklärungen

Diese Variable bezieht sich nur auf allgemeine Angaben bezüglich Erklärungen, die nicht einer der folgenden Variablen zu Erklärungen zugeordnet werden können: Erklärung von Testergebnissen (v_erkl_test), Diagnose (v_erkl_diag), Therapiemaßnahmen (v_erkl_therapie) oder Tipps (v_erkl_tipp). Die Codierung erfolgt auch, wenn Angaben darüber gemacht werden, ob der/die Mediziner:in grundsätzlich Erklärungen abgegeben hat, ohne Angaben über Verständlichkeit (3 = Erklärung verständlich).

Variablenname:
v_erkl_allg

Codierung:
1 = Erklärung nicht verständlich
2 = Erklärung nur teilweise verständlich
3 = Erklärung verständlich
4 = Erklärung sehr gut verständlich

**Beispiel**	**Codierung**
…ich habe die Erklärungen einfach nicht verstanden.	Es wird die Ausprägung **Erklärung nicht verständlich** (1) codiert.<br>Die Erklärungen wurden nicht/in keinster Weise verstanden.

…ohne wirkliche Erklärungen...	Es wird die Ausprägung **Erklärung nur teilweise verständlich** (2) codiert.<br>Erklärungen wurden grundsätzlich verstanden, zeigt sich aber eine negative Tendenz im Vergleich zu Erklärung verstanden, ist diese Ausprägung zu codieren.
…der/die Arzt/Ärztin hat mir alles verständlich erklärt.	Es wird die Ausprägung **Erklärung verständlich** (3) codiert.<br>Die Erklärungen wurden verstanden. Diese Ausprägung ist auch zu codieren, wenn generell die Angabe gemacht wurde, dass Erklärungen abgegeben wurden, ohne zusätzliche Angaben, ob diese verstanden wurden (es wird davon ausgegangen, dass die Erklärungen verstanden wurden, da bei Nicht-Verstehen, dies angeführt werden würde).
Kommunikation mit Patient:in ist sehr verständlich und ausführlich.<br>…erklärt so, dass auch Nicht-Medizin Absolvent:innen diese verstehen können.	Es wird die Ausprägung **Erklärung sehr gut verständlich** (4) codiert.<br>Erklärungen wurden verstanden mit zusätzlicher positiver Tendenz im Vergleich zu Erklärung verständlich.

7.2 Erklärung Testergebnisse

Diese Variable bezieht sich nur auf die Erklärung der Testergebnisse. Allgemeine Erklärungen (v_erkl_allg), Erklärung zu Diagnose (v_erkl_diag), Therapiemaßnahmen (v_erkl_therapie) sind hier nicht zu codieren.

<u>Variablenname:</u>
v_erkl_test

<u>Codierung:</u>
1 = Erklärung nicht verständlich
2 = Erklärung nur teilweise verständlich
3 = Erklärung verständlich
4 = Erklärung sehr gut verständlich

Beispiel	Codierung
Sie/Er schien in Eile zu sein denn weder meine aktuellen EKG noch meine Blutdruckbefunde wurden besprochen.	Es wird die Ausprägung **Erklärung nicht verständlich** (1) codiert.<br>Die Erklärungen wurden nicht/in keinster Weise verstanden oder Testergebnisse gar nicht erklärt.
…die Testergebnisse wurden mir zwar erklärt, was sie aber genau bedeuten nicht.	Es wird die Ausprägung **Erklärung nur teilweise verständlich** (2) codiert.<br>Erklärungen wurden grundsätzlich verstanden, zeigt sich aber eine negative Tendenz im Vergleich zu Erklärung verstanden, ist diese Ausprägung zu codieren.
…und hat mir alle SDWerte verständlich erklärt. Die anschließende Kommunikation über die Werte hat einwandfrei funktioniert.	Es wird die Ausprägung **Erklärung verständlich** (3) codiert.<br>Die Erklärungen wurden verstanden ohne zusätzliche positive oder negative Angaben.
..., dass ich es auch als Laie verstehen konnte.<br>Klare, sehr gut verständliche Erklärung.	Es wird die Ausprägung **Erklärung sehr gut verständlich** (4) codiert.<br>Erklärungen wurden verstanden mit zusätzlicher positiver Tendenz im Vergleich zu Erklärung verständlich.

7.3 Erklärung Diagnose

Diese Variable bezieht sich nur auf die Erklärung der Diagnose. Allgemeine Erklärungen (v_erkl_allg), Erklärung zu Testergebnissen (v_erkl_test), Therapiemaßnahmen (v_erkl_therapie) sind hier nicht zu codieren.

<u>Variablenname:</u>
v_erkl_diag

<u>Codierung:</u>
1 = Erklärung nicht verständlich
2 = Erklärung nur teilweise verständlich
3 = Erklärung verständlich
4 = Erklärung sehr gut verständlich

Beispiel	Codierung
…ärztliche Diagnose und Therapievorschläge habe ich absolut nicht verstanden.	Es wird die Ausprägung **Erklärung nicht verständlich** (1) codiert.<br>Die Erklärungen wurden nicht/in keinster Weise verstanden oder die Diagnose nicht erklärt.
…die Diagnose wurde mir zwar erklärt aber um sie wirklich zu verstehen, hätte ich mehr Hintergrundinformationen benötigt.	Es wird die Ausprägung **Erklärung nur teilweise verständlich** (2) codiert.<br>Erklärungen wurden grundsätzlich verstanden, zeigt sich aber eine negative Tendenz im Vergleich zu Erklärung verstanden, ist diese Ausprägung zu codieren.
Die Diagnose wurde verständlich erklärt.	Es wird die Ausprägung **Erklärung verständlich** (3) codiert.<br>Die Erklärungen wurden verstanden ohne zusätzliche positive oder negative Angaben.
..., dass ich es auch als Laie verstehen konnte.<br>Klare, sehr gut verständliche Erklärung.	Es wird die Ausprägung **Erklärung sehr gut verständlich** (4) codiert.<br>Erklärungen wurden verstanden mit zusätzlicher positiver Tendenz im Vergleich zu Erklärung verständlich.

7.4 Erklärung Therapie

Diese Variable bezieht sich nur auf die Erklärung Therapiemaßnahmen. Allgemeine Erklärungen (v_erkl_allg), Erklärung zu Testergebnissen (v_erkl_test) oder zur Diagnose (v_erkl_diag) sind hier nicht zu codieren.

Variablenname:
v_erkl_therapie

Codierung:
1 = Erklärung nicht verständlich
2 = Erklärung nur teilweise verständlich
3 = Erklärung verständlich
4 = Erklärung sehr gut verständlich

Beispiel	Codierung
…ärztliche Diagnose und Therapievorschläge habe ich absolut nicht verstanden.<br>Den Aufklärungsbogen musste ich unterschreiben, ohne aufgeklärt worden zu sein.	Es wird die Ausprägung **Erklärung nicht verständlich** (1) codiert.<br>Die Erklärungen wurden nicht/in keinster Weise verstanden oder die Therapiemaßnahmen nicht erklärt.
…ich habe die Erklärung zur Therapie nur halb verstanden und es wurde auch auf meine Fragen nicht eingegangen.	Es wird die Ausprägung **Erklärung nur teilweise verständlich** (2) codiert.<br>Erklärungen wurden grundsätzlich verstanden, zeigt sich aber eine negative Tendenz im Vergleich zu Erklärung verstanden, ist diese Ausprägung zu codieren.
…die Therapie wurde mir verständlich erklärt.	Es wird die Ausprägung **Erklärung verständlich** (3) codiert.<br>Die Erklärungen wurden verstanden ohne zusätzliche positive oder negative Angaben.
..., dass ich es auch als Laie verstehen konnte.<br>Klare, sehr gut verständliche Erklärung.	Es wird die Ausprägung **Erklärung sehr gut verständlich** (4) codiert.<br>Erklärungen wurden verstanden mit zusätzlicher positiver Tendenz im Vergleich zu Erklärung verständlich.

7.5 Tipps

Diese Variable enthält Erklärungen/Tipps/Empfehlungen/Rat für die persönliche Situation/Erkrankung/Verhalten des/der Patient:in. Es wird nicht unterschieden, ob es sich um Informationen über gesundheitsförderliche Verhaltensweisen oder speziell auf eine Erkrankung abzielende Erklärungen handelt.

Variablenname:
v_erkl_tipp

Codierung:
1 = gar nicht
2 = eher weniger
3 = eher
4 = sehr

**Beispiel**	**Codierung**
…ich wollte wissen, wie ich meine gesundheitliche Situation verbessern kann. Der/Die Ärztin ist darauf überhaupt nicht eingegangen.	Es wird die Ausprägung **gar nicht** (1) codiert.<br>Es wurden keine Tipps/Empfehlungen/Rat ausgesprochen.
Ich habe zwar Tipps erhalten, es war aber nichts dabei, was ich nicht ohnehin schon wusste.	Es wird die Ausprägung **eher weniger** (2) codiert.<br>Es wurden grundsätzlich Tipps/Empfehlungen/Rat ausgesprochen, diese bezogen sich allerdings nicht individuelle auf den/die Patient:in oder haben dem/der Patient:in nicht geholfen.
...gibt Tipps...	Es wird die Ausprägung **eher** (3) codiert.<br>Individuelle Tipps/Empfehlungen/Rat wurden ausgesprochen ohne positive oder negative Tendenz.
…, dabei stand sie/er mir mit nützlichen Tipps stets zur Seite.	Es wird die Ausprägung **sehr** (4) codiert.<br>Individuelle Tipps/Empfehlungen/Rat wurden ausgesprochen mit positiver Tendenz (gut, sehr gut).

7.6 Berücksichtigung der Bedürfnisse

Diese Variable beinhaltet, ob der/die Ärztin auf Anliegen/Interessen/Wünsche eingeht. Nicht beinhaltet sind hier Sorgen/Ängste (v_bez_angst). Zusätzliche Signalwörter für diese Variable: aufmerksam, bemüht, zuvorkommend; Nicht: Menschlich - wird unter der Variable v_bez_empathie codiert.

Variablenname:

v_bed_e

Codierung:

1 = gar nicht

2 = eher weniger

3 = eher

4 = sehr

**Beispiel**	**Codierung**
Es wurde nicht auf meine Anliegen eingegangen…<br>…nimmt sich nicht mal Zeit für meine Anliegen.	Es wird die Ausprägung **gar nicht** (1) codiert.<br>Die Bedürfnisse wurden nicht/in keinster Weise berücksichtigt.

…lässt einen kaum ausreden/antworten, will schnell weiter.	Es wird die Ausprägung **eher weniger** (2) codiert.<br>Patient:in hat das Gefühl, dass Bedürfnisse wahrgenommen, allerdings nicht berücksichtigt oder darauf eingegangen wurde.
…ist auf meine Bedürfnisse eingegangen.<br>…war zuvorkommend.	Es wird die Ausprägung **eher** (3) codiert.<br>Auf die Bedürfnisse wurde eingegangen ohne positive oder negative Tendenz.
…ist auf meine Anliegen sehr genau eingegangen.<br>…war sehr aufmerksam.	Es wird die Ausprägung **sehr** (4) codiert.<br>Die Bedürfnisse wurden vollkommen berücksichtigt und sehr ausführlich darauf eingegangen.

7.7 Empathie

Diese Variable bezieht sich auf die subjektive Wahrnehmung der Empathie des/der Arztes/Ärztin von dem/der Patient:in. Dies beinhaltet auch Mitgefühl, Taktgefühl, Feinfühligkeit, Menschlichkeit. Zusätzliche Signalwörter: hilfsbereit, menschlich, herzlich.

Variablenname:
v_bez_empathie

Codierung:
1 = gar nicht
2 = eher weniger
3 = eher
4 = sehr

**Beispiele**	**Codierung**
…fand den/die Arzt/Ärztin nicht einfühlsam und nicht hilfsbereit.<br>Persönliche Anteilnahme kann man sicher nicht erwarten ...	Es wird die Ausprägung **gar nicht** (1) codiert.<br>Der/die Arzt/Ärztin wurde als nicht empathisch empfunden oder hat keine Empathie gezeigt.
…dieser/diese Arzt/Ärztin ist nicht sehr empathisch…	Es wird die Ausprägung **eher weniger** (2) codiert.<br>Der/die Arzt/Ärztin wurde als eher nicht empathisch empfunden oder hat wenig Empathie gezeigt.

Professionell und empathisch kümmerte er/sie sich um mich.	Es wird die Ausprägung **eher** (3) codiert.<br>Der/die Arzt/Ärztin wurde als empathisch empfunden oder hat Empathie gezeigt.
Auf etwaige Fragen geht sie/er mit großem Einfühlungsvermögen und Geduld ein…<br>...ist sehr bemüht und empathisch.	Es wird die Ausprägung **sehr** (4) codiert.<br>Der/die Arzt/Ärztin wurde als sehr empathisch empfunden oder hat sehr hohe Empathie gezeigt.

7.8 Vertrauen

Diese Variable beinhaltet, ob der/die Patient:in den/die Arzt/Ärztin vertrauensvoll gefunden oder ihm/ihr vertraut hat. Auch alle Angaben über Vertrauen aufzubauen oder das Gefühl gut aufgehoben zu sein werden hier miteinbezogen.

Variablenname:
v_bez_vertrau

Codierung:
0 = Nein
1 = Ja

**Beispiele**	**Codierung**
…ich konnte kein Vertrauen aufbauen.	Es wird die Ausprägung **Nein** (0) codiert.<br>Arzt/Ärztin war nicht vertrauensvoll, Vertrauen konnte nicht aufgebaut werden.
Er/Sie hat sehr schnell ein persönliches Vertrauensverhältnis aufgebaut.	Es wird die Ausprägung **Ja** (1) codiert.<br>Arzt/Ärztin war vertrauensvoll, Vertrauen konnte aufgebaut werden. Patient:in vertraut dem/der Arzt/Ärztin.

7.9 Partnerschaftliches Verhältnis

Diese Variable beinhaltet, ob der/die Arzt/Ärztin den/die Patient:in als Partner:in in der Behandlung gesehen wird. Auch wie mit dem/der Patient:in (von oben herab oder auf Augenhöhe) kommuniziert wurde, wird hier berücksichtigt.

Variablenname:
v_bez_partner

<u>Codierung:</u>

0 = Nein

1 = Ja

Beispiele	Codierung
Leider ist mir seine/ihre Gesprächsführung sehr von oben herab vorgekommen…	Es wird die Ausprägung **Nein** (0) codiert.<br>Die Beziehung/Kommunikation war nicht partnerschaftlich / war von oben herab.
...bespricht alles auf Augenhöhe…<br>...hat nie von oben herab mit mir gesprochen.	Es wird die Ausprägung **Ja** (1) codiert.<br>Die Beziehung/Kommunikation war partnerschaftlich/ auf Augenhöhe.

7.10 Persönliche Situation

Diese Variable bezieht sich auf das Umfeld des/der Patient:in, wie beispielsweise das familiäre, persönliche, gesundheitliche Umfeld - eine ganzheitliche Perspektive auf den/die Patient:in.

<u>Variablenname:</u>

v_bez_pers

<u>Codierung:</u>

0 = Nein

1 = Ja

Beispiele	Codierung
…fixiert sich nur auf das eine Problem und hat meine persönliche Situation überhaupt nicht berücksichtigt.	Es wird die Ausprägung **Nein** (0) codiert.<br>Die individuelle Situation/Rahmenbedingungen/Gesundheitszustand wurde nicht berücksichtigt.
…sieht die Patient:innen ganzheitlich.<br>Auch achtet er/sie darauf, dass ich alle, nicht in seinen/ihren Bereich fallenden, regelmäßig fälligen Untersuchungen auch wirklich mache.	Es wird die Ausprägung **Ja** (1) codiert.<br>Die individuelle Situation/Rahmenbedingungen/Gesundheitszustand wurde berücksichtigt.

7.11 Meinung des/der Patient:in

Diese Variable beinhaltet, ob der/die Arzt/Ärztin den/die Patient:in aktiv nach der Meinung über Behandlungsmöglichkeiten/weiteres Vorgehen gefragt wurde.

Variablenname:
v_komm_meinung

Codierung:
0 = Nein
1 = Ja

Beispiele	Codierung
…ich hätte gerne eine andere Behandlung gehabt aber der/die Arzt/Ärztin hat mich nicht gefragt was ich möchte.	Es wird die Ausprägung **Nein** (0) codiert.<br>Es wurde nicht nach der Meinung des/der Patient:in gefragt.
…ich hatte sogar die Möglichkeit meine Meinung zu der Behandlung zu sagen.	Es wird die Ausprägung **Ja** (1) codiert.<br>Die Meinung des/der Patient:in wurde miteinbezogen.

7.12 Rückfragen Verständnis seitens Mediziner:in

Diese Variable ist bereits in der Kategorie Gesundheitskompetenz angeführt, ist allerdings ebenfalls für die Kategorie Selbstmanagement von Bedeutung, wodurch die Variable ebenfalls hier aufgelistet wird. Die genaue Beschreibung erfolgt allerdings unter Punkt 5.6. Die Codierung wird nur einmal vorgenommen.

8 Kategorie Kommunikation

Die einzelnen Variablen beziehen sich auf relevante Aspekte zur Kommunikation in der Arzt-Patient-Beziehung und wurden aus der Literatur abgeleitet.

8.1 Fragen stellen

Diese Variable beinhaltet, ob der/die Patient:in im Gespräch mit dem/der Arzt/Ärztin die Möglichkeit bekommen haben ihre Fragen zu stellen. Nicht berücksichtigt wird hier, ob die Fragen auch beantwortet wurden (v_f_beant). Sobald beschrieben wird, dass die gestellten Fragen beantwortet wurden, wird neben der Variable v_f_beant auch diese Variable codiert.

Variablenname:
v_f_stellen

Codierung:
0 = Nein
1 = Ja

**Beispiele**	**Codierung**
…lässt einen nicht reden/antworten, will schnell weiter.	Es wird die Ausprägung **Nein** (0) codiert.<br>Fragen konnten nicht gestellt werden.
...gibt auch beim dritten Mal nachfragen noch immer ausführliche Antworten.	Es wird die Ausprägung **Nein** (0) codiert.<br>Fragen konnten im Gespräch gestellt werden.

8.2 Beantwortung der Fragen

Diese Variable beinhaltet, ob der/die Arzt/Ärztin die Fragen beantwortet hat. Wenn Angaben über die Beantwortung der gestellten Fragen gemacht werden, ist auch die Variable v_f_stellen zu codieren.

Variablenname:
v_f_beant

<u>Codierung:</u>

1 = gar nicht

2 = eher weniger

3 = eher

4 = sehr

**Beispiele**	**Codierung**
…hat bestehende Fragen ignoriert und ist nicht darauf eingegangen.	Es wird die Ausprägung **gar nicht** (1) codiert.<br>Der/die Arzt/Ärztin hat keine/nicht/keineswegs die gestellten Fragen beantwortet.
...doch, wenn man etwas genauer wissen möchte, bekommt man motzige Antworten.	Es wird die Ausprägung **eher weniger** (2) codiert.<br>Fragen sind zwar beantwortet worden, allerdings nicht zur Zufriedenheit oder nicht ausreichend.
…ein(e) Arzt/Ärztin, der/die alle Fragen des/der Patient:in beantwortet.	Es wird die Ausprägung **eher** (3) codiert.<br>Fragen wurden beantwortet ohne positive oder negative Tendenz.
…gibt auch beim dritten Mal nachfragen noch immer ausführliche Antwort.<br>Mir wurde alles erklärt und jede Frage ausführlich beantwortet.	Es wird die Ausprägung **sehr** (4) codiert.<br>Fragen wurden beantwortet, mit zusätzlich positiven Angaben (z. B. äußerst ausführlich, sehr genau, sehr umfassend).

8.3 Eingehen auf Ängste

Diese Variable ist bereits in der Kategorie Shared decision making angeführt, ist allerdings ebenfalls in der Kommunikation im Arzt-Patient-Gespräch von Bedeutung, wodurch die Variable ebenfalls hier aufgelistet wird. Die genaue Beschreibung erfolgt allerdings unter Punkt 6.6. Die Codierung wird nur einmal vorgenommen.

8.4 Zuhören des/der Arztes/Ärztin

Diese Variable ist bereits in der Kategorie Gesundheitskompetenz angeführt, ist allerdings ebenfalls in der Kommunikation im Arzt-Patient-Gespräch von Bedeutung, wodurch die Variable ebenfalls hier aufgelistet wird. Die genaue Beschreibung erfolgt allerdings unter Punkt 5.3. Die Codierung wird nur einmal vorgenommen.

8.5 Zeigen von Verständnis

Diese Variable beinhaltet, ob der/die Patient:in das Gefühl hatte, dass der/die Arzt Verständnis gegenüber der Situation/Bedenken gezeigt hat. Nicht gemeint ist hier die Empathie (v_bez_empathie), auf den/die Patient:in eingehen (v_bez_e) oder das Zeigen von Respekt (v_bez_respekt).

<u>Variablenname:</u>
v_bez_verst

<u>Codierung:</u>
1 = gar nicht
2 = eher weniger
3 = eher
4 = sehr

**Beispiele**	**Codierung**
…zeigte keinerlei Verständnis für meine Bedenken und Situation.	Es wird die Ausprägung **gar nicht** (1) codiert.<br>Es wurde gegenüber dem/der Patient:in kein Verständnis gezeigt.
…zeigte nicht wirklich Verständnis für meine Beschwerden.	Es wird die Ausprägung **eher weniger** (2) codiert.<br>Es wurde gegenüber dem/der Patient:in wenig Verständnis gezeigt.
...hat Verständnis, …	Es wird die Ausprägung **eher** (3) codiert.<br>Es wurde gegenüber dem/der Patient:in Verständnis gezeigt ohne positive oder negative Tendenz.
...ist sehr verständnisvoll und geht auf meine Ängste ein.	Es wird die Ausprägung **sehr** (4) codiert.<br>Der/die Arzt/Ärztin war gegenüber dem/der Patient:in sehr verständnisvoll.

8.6 Zeigen von Respekt

Diese Variable beinhaltet, ob sich der/die Patient:in respektvoll behandelt gefühlt hat. Nicht hinzu zählt die Empathie (v_bez_empathie) und das entgegengebrachte Verständnis (v_bez_verst).

Variablenname:

v_bez_respekt

Codierung:

0 = Nein

1 = Ja

Beispiele	Codierung
…ich fühlte mich nicht sehr respektvoll behandelt.	Es wird die Ausprägung **Nein** (0) codiert.<br>Der/die Arzt/Ärztin zeigte keinen Respekt gegenüber dem/der Patient:in.
…sehr einfühlsam, sympathisch und respektvoll.	Es wird die Ausprägung **Ja** (1) codiert.<br>Der/die Arzt/Ärztin zeigte Respekt gegenüber dem/der Patient:in.

8.7 Ehrlichkeit

Diese Variable beinhaltet, ob der/die Patient:in das Gefühl hatten, dass der/die Arzt/Ärztin alle Informationen ehrlich vermittelt und keine Informationen vorenthalten hat.

Variablenname:

v_bez_ehrlich

Codierung:

0 = Nein

1 = Ja

Beispiele	Codierung
Man hatte das Gefühl, nicht alle Informationen zu bekommen und dass etwas verschwiegen wurde.	Es wird die Ausprägung **Nein** (0) codiert.<br>Es wurden nicht alle Informationen weitergegeben; Arzt/Ärztin war nicht ehrlich.
…nimmt kein Blatt vor den Mund, auch wenn es mal schlechte Befunde gibt.	Es wird die Ausprägung **Ja** (1) codiert.<br>Es wurden keine Informationen vorenthalten; Arzt/Ärztin war ehrlich.

8.8 Vertrauen

Diese Variable ist bereits in der Kategorie Selbstmanagement angeführt, ist allerdings ebenfalls in der Kommunikation im Arzt-Patient-Gespräch von Bedeutung, wodurch die Variable ebenfalls hier aufgelistet wird. Die genaue Beschreibung erfolgt allerdings unter Punkt 7.8. Die Codierung wird nur einmal vorgenommen.

8.9 Ernst nehmen

Diese Variable beinhaltet, ob sich der/die Patient:in im Allgemeinen während der Behandlung/Gespräch ernst genommen gefühlt hat. Nicht beinhaltet ist hier die Spezifizierung Empathie (v_bez_empathie), der respektvolle Umgang (v_bez_respekt) oder das Zeigen von Verständnis (v_bez_verst).

Variablenname:
v_bez_ernst

Codierung:
0 = Nein
1 = Ja

**Beispiele**	**Codierung**
Der/Die Arzt/Ärztin hat mich nicht sehr ernst genommen.	Es wird die Ausprägung **Nein** (0) codiert.<br>Der/die Patient:in wurde nicht oder nur teilweise ernst genommen.
...man fühlt sich auch mit den kleinsten Beschwerden sehr ernst genommen.	Es wird die Ausprägung **Ja** (1) codiert.<br>Der/die Patient:in wurde ernst genommen.

9 Atmosphäre

Diese Variable wird dann codiert, wenn der/die Patient:in Angaben über die allgemeine Atmosphäre gemacht hat.

Variablenname:

v_umgebung

Codierung:

1 = Sehr angespannte Atmosphäre

2 = Gestresste Atmosphäre

3 = Angenehme Atmosphäre

4 = Ruhige und entspannte Atmosphäre

**Beispiele**	**Codierung**
…die gesamte Atmosphäre empfand ich als sehr unentspannt.	Es wird die Ausprägung **sehr angespannte Atmosphäre** (1) codiert.<br>Das gesamte Umfeld/Besuch hat auf den/die Patient:in sehr angespannt gewirkt.
...gestresst und unpersönlich.	Es wird die Ausprägung **gestresste Atmosphäre** (2) codiert.<br>Der Gesamteindruck war sehr gestresst - explizit auf stressig empfundene Situation.
...angenehme Atmosphäre im Wartebereich und im Gespräch mit dem/der Arzt/Ärztin. Schönes Ambiente. Gutes Klima.	Es wird die Ausprägung **angenehme Atmosphäre** (3) codiert.<br>Das gesamte Umfeld/der Besuch wurde sehr angenehm/positiv empfunden - nicht ruhig und entspannt.
In der Ordination herrscht eine entspannte, aber doch konzentrierte Atmosphäre.	Es wird die Ausprägung **ruhige und entspannte Atmosphäre** (4) codiert.<br>Der Gesamteindruck war sehr entspannt und ruhig - nicht allgemein angenehm.

10 Keine Angabe zu Patient Empowerment

Diese Variable wird immer dann codiert, wenn keine der inhaltlichen Variablen zu Patient Empowerment (Variablen der Kapitel 4-8) im Kommentartext codiert werden können. Konnte eine Variable zu Patient Empowerment codiert werden, erfolgt zu dieser Variable keine Codierung.

<u>Variablenname:</u>
v_pe_k

<u>Codierung:</u>
1 = keine Angabe zu Patient Empowerment

**Beispiele**
Meine Erwartungen wurden optimal erfüllt!
Tolles Team, schöne Praxis, kompetente(r) Arzt/Ärztin! Hab schnell einen Termin bekommen.
Ich habe bei Dr. … ein großes Blutbild gemacht. Die Blutabnahme ging so schnell, habe nicht mal gemerkt, dass er/sie die Ampullen gewechselt hat. Oft wurde bei mir verstochen oder ich hatte große Schmerzen - nicht hier. Kann ich nur empfehlen!
